# Pyruvate dehydrogenase kinase regulates vascular inflammation in atherosclerosis and increases cardiovascular risk

**DOI:** 10.1093/cvr/cvad038

**Published:** 2023-03-02

**Authors:** Maria J Forteza, Martin Berg, Andreas Edsfeldt, Jangming Sun, Roland Baumgartner, Ilona Kareinen, Felipe Beccaria Casagrande, Ulf Hedin, Song Zhang, Ivan Vuckovic, Petras P Dzeja, Konstantinos A Polyzos, Anton Gisterå, Mette Trauelsen, Thue W Schwartz, Lea Dib, Joerg Herrmann, Claudia Monaco, Ljubica Matic, Isabel Gonçalves, Daniel F J Ketelhuth

**Affiliations:** Center for Molecular Medicine, Department of Medicine, Solna, Karolinska University Hospital, Karolinska Instutet, BioClinicum, Solnavägen 30, Solna, 17 164, Stockholm, Sweden; Center for Molecular Medicine, Department of Medicine, Solna, Karolinska University Hospital, Karolinska Instutet, BioClinicum, Solnavägen 30, Solna, 17 164, Stockholm, Sweden; Cardiovascular Research Translational Studies, Clinical Research Centre, Clinical Sciences Malmö, Lund University, Jan Waldenströms gata 35, 20 502, Malmö, Sweden; Department of Cardiology, Skåne University Hospital, Carl-Bertil Laurells gata 9, 21 428, Malmö, Sweden; Wallenberg Center for Molecular Medicine, Lund University, Jan Waldenströms gata 35, 20 502, Malmö, Sweden; Cardiovascular Research Translational Studies, Clinical Research Centre, Clinical Sciences Malmö, Lund University, Jan Waldenströms gata 35, 20 502, Malmö, Sweden; Department of Cardiology, Skåne University Hospital, Carl-Bertil Laurells gata 9, 21 428, Malmö, Sweden; Center for Molecular Medicine, Department of Medicine, Solna, Karolinska University Hospital, Karolinska Instutet, BioClinicum, Solnavägen 30, Solna, 17 164, Stockholm, Sweden; Center for Molecular Medicine, Department of Medicine, Solna, Karolinska University Hospital, Karolinska Instutet, BioClinicum, Solnavägen 30, Solna, 17 164, Stockholm, Sweden; Center for Molecular Medicine, Department of Medicine, Solna, Karolinska University Hospital, Karolinska Instutet, BioClinicum, Solnavägen 30, Solna, 17 164, Stockholm, Sweden; Department of Molecular Medicine and Surgery, Karolinska University Hospital, Karolinska Institutet, BioClinicum, Solnavägen 30, Solna, 17 164, Stockholm, Sweden; Mayo Clinic Metabolomics Core, Mayo Clinic, 200, First St. SW Rochester, MN 55905, USA; Department of Cardiovascular Medicine, Mayo Clinic, 200, First St. SW Rochester, MN 55905, USA; Mayo Clinic Metabolomics Core, Mayo Clinic, 200, First St. SW Rochester, MN 55905, USA; Department of Cardiovascular Medicine, Mayo Clinic, 200, First St. SW Rochester, MN 55905, USA; Center for Molecular Medicine, Department of Medicine, Solna, Karolinska University Hospital, Karolinska Instutet, BioClinicum, Solnavägen 30, Solna, 17 164, Stockholm, Sweden; Center for Molecular Medicine, Department of Medicine, Solna, Karolinska University Hospital, Karolinska Instutet, BioClinicum, Solnavägen 30, Solna, 17 164, Stockholm, Sweden; Section for Metabolic Receptology, Novo Nordisk Foundation Center for Basic Metabolic Research, University of Copenhagen, Blegdamsvej 3A, 2200, Copenhagen, Denmark; Section for Metabolic Receptology, Novo Nordisk Foundation Center for Basic Metabolic Research, University of Copenhagen, Blegdamsvej 3A, 2200, Copenhagen, Denmark; Kennedy Institute of Rheumatology, University of Oxford, Roosevelt Dr, Headington, Oxford OX3 7FY, UK; Department of Cardiovascular Medicine, Mayo Clinic, 200, First St. SW Rochester, MN 55905, USA; Kennedy Institute of Rheumatology, University of Oxford, Roosevelt Dr, Headington, Oxford OX3 7FY, UK; Department of Molecular Medicine and Surgery, Karolinska University Hospital, Karolinska Institutet, BioClinicum, Solnavägen 30, Solna, 17 164, Stockholm, Sweden; Cardiovascular Research Translational Studies, Clinical Research Centre, Clinical Sciences Malmö, Lund University, Jan Waldenströms gata 35, 20 502, Malmö, Sweden; Department of Cardiology, Skåne University Hospital, Carl-Bertil Laurells gata 9, 21 428, Malmö, Sweden; Center for Molecular Medicine, Department of Medicine, Solna, Karolinska University Hospital, Karolinska Instutet, BioClinicum, Solnavägen 30, Solna, 17 164, Stockholm, Sweden; Department of Cardiovascular and Renal Research, Institute of Molecular Medicine, University of Southern Denmark, J.B. Winsløws vej 21, 5000 Odense, Denmark

**Keywords:** atherosclerosis, inflammation, PDK, CVD, immunometabolism

## Abstract

**Aims:**

Recent studies have revealed a close connection between cellular metabolism and the chronic inflammatory process of atherosclerosis. While the link between systemic metabolism and atherosclerosis is well established, the implications of altered metabolism in the artery wall are less understood. Pyruvate dehydrogenase kinase (PDK)-dependent inhibition of pyruvate dehydrogenase (PDH) has been identified as a major metabolic step regulating inflammation. Whether the PDK/PDH axis plays a role in vascular inflammation and atherosclerotic cardiovascular disease remains unclear.

**Methods and results:**

Gene profiling of human atherosclerotic plaques revealed a strong correlation between *PDK1* and *PDK4* transcript levels and the expression of pro-inflammatory and destabilizing genes. Remarkably, the *PDK1* and *PDK4* expression correlated with a more vulnerable plaque phenotype, and *PDK1* expression was found to predict future major adverse cardiovascular events. Using the small-molecule PDK inhibitor dichloroacetate (DCA) that restores arterial PDH activity, we demonstrated that the PDK/PDH axis is a major immunometabolic pathway, regulating immune cell polarization, plaque development, and fibrous cap formation in *Apoe*−*/*− mice. Surprisingly, we discovered that DCA regulates succinate release and mitigates its GPR91-dependent signals promoting NLRP3 inflammasome activation and IL-1β secretion by macrophages in the plaque.

**Conclusions:**

We have demonstrated for the first time that the PDK/PDH axis is associated with vascular inflammation in humans and particularly that the *PDK1* isozyme is associated with more severe disease and could predict secondary cardiovascular events. Moreover, we demonstrate that targeting the PDK/PDH axis with DCA skews the immune system, inhibits vascular inflammation and atherogenesis, and promotes plaque stability features in *Apoe*−/− mice. These results point toward a promising treatment to combat atherosclerosis.

**Time of primary review: 36 days**

**Translational perspective**

Certain alterations of the intracellular metabolism of immune cells have been proposed to exacerbate inflammation and promote different diseases. Our work shows that the skewing of the pyruvate dehydrogenase kinase/pyruvate dehydrogenase (PDK/PDH) metabolic axis, a gatekeeping step linking glycolysis to the tricarboxylic acid cycle (TCA), in the human plaque increases the risk of future cardiovascular events. Moreover, our findings indicate that inhibition of PDK activity, particularly on macrophages, could be used as an immunometabolic approach to combat vascular inflammation, prevent atherogenesis, and promote plaque stability.

## 1. Introduction

Myocardial infarction and stroke, largely due to atherosclerosis, remain the leading causes of death worldwide, and their incidence is still increasing in developing countries.^1^ Atherosclerosis is a chronic inflammatory disease characterized by pathological responses of immune cells that promote atheroma formation in large- and medium-sized arteries. Particularly in recent decades, compelling data from basic and clinical research indicate that the balance between pro- and anti-inflammatory responses of the immune system can determine the rate of disease progression, as well as plaque stability.^2^

Recent studies looking for culprit regulatory mechanisms of inflammation have revealed a close connection between cellular metabolism and immune cell function. Pro-inflammatory cells, including M1-like macrophages and effector T-helper (Th) 1 and Th17 cells, are likely to rely more on aerobic glycolysis for energy production—a phenomenon known as the Warburg effect, which has been described in tumour cells. Conversely, an increased mitochondrial oxidative metabolism has been associated with the anti-inflammatory responses of alternatively activated M2-like macrophages as well as regulatory T-cells (Tregs).^3^ Notably, the metabolic profile of immune cells is not static, and several studies indicate that the manipulation of macrophage and T-cell metabolism can be used to combat their most deleterious responses in the context of auto-immune and auto-inflammatory diseases.^4^

While the link between systemic metabolism, e.g. cholesterol and glucose, and atherosclerosis is undoubtedly very strong, the implications of altered metabolism in the artery wall are less understood.^5^ Recent metabolomic analysis of human carotid plaques revealed that in addition to increased glycolysis, which has been demonstrated by the positron emission tomography analysis of 18F-fluorodeoxyglucose uptake,^6^ unstable plaques present elevated amino acid utilization and decreased fatty acid oxidation,^7^ indicating a quite complex tissue metabolic status associated with vascular inflammation. Whether targeting the metabolism of immune cells can influence cardiovascular disease (CVD) remains largely unknown.

The mitochondrial pyruvate dehydrogenase complex (PDHc), which is composed of pyruvate dehydrogenase (PDH), dihydrolipoamide S-acetyltransferase, and dihydrolipoamide dehydrogenase, is a metabolic gatekeeping step linking glycolysis and gluconeogenesis with the tricarboxylic acid cycle (TCA) and subsequent oxidative phosphorylation.^8^ PDHc activity is inhibited primarily by the phosphorylation of specific serine residues on PDH, catalyzed by four pyruvate dehydrogenase kinases (*PDK1–4*).^9^ In general, it has been indicated that PDK isoforms exhibit a tissue-specific expression pattern, e.g. *PDK1* and *PDK4* are highly expressed in the heart, pancreatic islets, and skeletal muscles and *PDK3* is especially present in the testis, kidney, and brain, while *PDK2* seemed present at a more widespread expression profile.^10^ As consequence of overexpressing PDK isoenzymes, decreased PDH activity has been associated with M1 and Th17 polarization and the exacerbation of inflammatory processes in different diseases, including Alzheimer’s disease, multiple sclerosis, and different types of cancer.^11,12^ The role of the PDK/PDH axis in vascular inflammation and CVD risk has never been studied.

In the present study, we performed a gene expression profile analysis of human carotid endarterectomy specimens to investigate the associations between the expression of PDK isoenzymes and vascular inflammation and plaque vulnerability, as well as associations with the development of cerebrovascular-related symptoms, and future major adverse cardio- and cerebrovascular events (MACCE) following up endarterectomy. Together with experiments *in vitro* and the targeting of PDKs *in vivo* in *Apoe*−*/−* mice, we demonstrate that the PDK/PDH axis is a master immunometabolic regulator of immune cell polarization, plaque development, and fibrous cap formation. Altogether, our results indicate promising new strategies to prevent and treat atherosclerotic CVDs.

## 2. Methods

An extended description of methods is provided in the Supplementary Material online.

### 2.1 Human atherosclerotic plaque material and data: *biobanks*

#### 2.1.1 Carotid Plaque Imaging Project biobank

All patients included in the Carotid Plaque Imaging Project (CPIP) biobank (Lund University, Malmö, Sweden) have undergone carotid endarterectomy due to carotid stenosis > 70% (verified by duplex ultrasound) and associated symptoms (stroke/TIA/amaurosis fugax) or due to carotid stenosis > 80% without associated symptoms (Lund University, Malmö, Sweden). The CPIP cohort demographics and details regarding sample collection, processing, and large-scale analyses have been previously described.^13^ Briefly, from the Swedish National Patient Register, the following ICD codes were used to define the endpoints of cardiovascular (CV) events in the survival analyses: G45.3, G45.9, G46, I21-22, I24.8–9, I25.1–2, I25.5–6, I25.8, I63.1–2, I63.3–5, I63.8–9, and I64.5; from the Swedish cause of death register, the following ICD-10 codes were used to define CV deaths: I10, I11.0, I11.9, I13.0, I13.2, I20.9, I25.9, I46.9, I48, I48.9, I49.0, I50.1, I50.9, I60.9, I61.9, I64, I69.3–4, I70.0, I70.2, I71.0–6, I71.8–9, I70.9, I72, I73.9, I74, I74.9, and I99. E78.5. Events occurring within 72 h post-surgery (carotid endarterectomy) were excluded as being procedure-related. If a patient suffered from multiple events, only the date of the first one was used in our survival analysis.

#### 2.1.2 Biobank of Karolinska Endarterectomies

Patients undergoing surgery for high-grade (>50% NASCET) carotid stenosis at the Department of Vascular Surgery, Karolinska University Hospital, Stockholm, Sweden, were consecutively enrolled in the study, and clinical data were recorded on admission. Carotid endarterectomies (carotid plaques) were collected at surgery and retained within the Biobank of Karolinska Endarterectomies (BiKE). The BiKE study cohort demographics and details regarding sample collection, processing, and large-scale analyses have been previously described.^14–16^

Informed consent was obtained from all subjects included in CPIP and BiKE. The investigations performed were approved by the local ethical committees and agreed with institutional guidelines and the principles outlined in the Declaration of Helsinki.

### 2.2 Animals and in vivo treatments

Twelve-week-old male *Apoe−/−* mice (Taconic Europe, Denmark) were randomized into three groups and treated with 1 mg/mL or 0.1 mg/mL DCA (Sigma-Aldrich, St. Louis, USA) diluted in drinking water (∼170 and 17 mg/kg/day, respectively) or with control tap water for 8 weeks. Treatment was delivered in standard autoclaved water bottles, protected from light, and changed twice per week. The DCA treatment was well tolerated, with no adverse clinical signs; DCA-treated mice drank approximately 5.1 ± 0.27 mL/day, similar to the consumption of water without drug drank by control mice, 5.4 ± 0.19 mL/day (*P* = 0.47). DCA doses were selected based on previous studies showing effects in the context of experimental autoimmune diseases.^17,18^ Concomitant to treatments, the mice were fed a western diet (0.15% cholesterol: R638 Lantmännen, Sweden) *ad libitum*. The mice were euthanized in a CO_2_ chamber at the end of treatments. For mechanistic investigations, only groups receiving 1 mg/mL (∼170 mg/kg/day) DCA and controls were used for comparisons. All animal experiments were conducted according to the guidelines of the Directive 2010/63/EU of the European Parliament on the protection of animals used for scientific purposes and approved by the Ethical Committee of the Northern Stockholm region board (N28-15).

### 2.3 Evaluation of the inflammasome activation in macrophages in vitro

To prime the NLRP3 inflammasome, 5 × 10^5^ BMDMs were pre-treated with 10 ng/mL LPS (Enzo Biochem, Farmingdale, NY, USA) for 4 h, followed by the addition of 1 mmol/L ATP (Sigma-Aldrich, St. Louis, IL, USA) for 1 h. Additionally, some cells were pre-treated with DCA to inactivate inflammasome activity for 30 min and then treated with LPS and/or ATP. Similarly, some cells were activated with 10 ng/mL LPS and then treated with DCA for 30 min before adding 1 mmol/L ATP for 1 h. In addition, some cells were treated with different concentrations (0, 5, or 10 mmol/L) of sodium succinate or with dimethyl succinate (Sigma-Aldrich, St. Louis, USA) with or without pertussis toxin (200 ng/mL, Sigma-Aldrich, St. Louis, USA) and with or without GPR91 antagonist validated at the CBMR Metabolic Receptology laboratory [cpmd0441 (IC_50_ = 2.63E − 06M); synthesized by SIA Enamine, Latvia] for 2 h prior to experiments. IL-1β production in the supernatants of cultures was analysed by ELISA (R&D Systems, MN, USA) according to the manufacturer’s instructions.

#### 2.3.1 Statistical analysis

A non-parametric Mann–Whitney *U* test was used for comparisons between two groups. Comparisons between two or more groups were performed using nonparametric Kruskal–Wallis ANOVA, followed by Dunn’s *post hoc* test. In all analyses, differences were considered significant at *P*-values < 0.05 (two-tailed). All statistical analyses of *in vivo* and *in vitro* experiments were performed using GraphPad Prism version 8.0 for Mac OS X (GraphPad Software Inc., La Jolla, USA).

For the survival analysis, all CPIP subjects included in the RNA sequencing (RNA-Seq) analysis were divided into tertiles based on *PDK1–4* gene expression. All CV events were registered during follow-up to explore the predictive role of *PDK1–4* gene expression. Events included [myocardial infarction, unstable angina, stroke, transient ischemic attack, amaurosis fugax, vascular interventions (including carotid endarterectomy/stenting and coronary artery bypass grafting/percutaneous coronary artery intervention), and CV death] were identified through the Swedish National Inpatient Health Register (2005–2013), and detailed ICD-10 codes have been described above. Data were analysed using SPSS 24.0 (IBM Corp., NY, USA). Spearman’s statistics were used for correlation analyses or otherwise specified in the figure legend. Kaplan–Meier curves and log-rank test were used for the survival analyses.

## 3. Results

### 3.1 The expression of *PDK1* and *PDK4* isoenzymes is associated with carotid atherosclerotic plaque vulnerability and *PDK1* expression predicts future cardiovascular events

**Figure 1:**
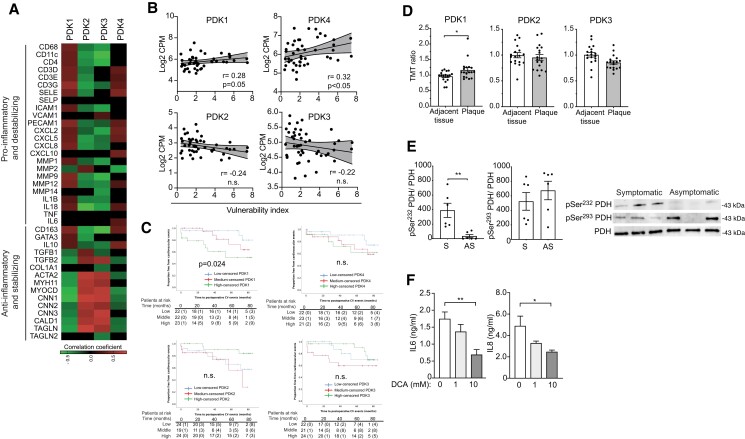
*PDK1* and *PDK4* positively correlate with inflammation and the vulnerability index in human atherosclerotic plaques, and *PDK1* is associated with the risk of future cardiovascular (CV) events. (*A*) Heat map of RNA-Seq gene expression data from human carotid plaques (CPIP biobank) showing correlations between the different PDK isoforms and genes related to inflammation or plaque stabilization. The genes were manually categorized as pro- inflammatory and plaque destabilizing or anti-inflammatory and plaque stabilizing. Black cells represent nonsignificant correlations or *R* = 0. Spearman correlations, corrected *P* < 0.05 was considered significant; *n* = 78. (*B*) Spearman correlation analysis between *PDK1–4* expression and the plaque vulnerability index on CPIP samples; *n* = 47. (*C*) Kaplan–Meier cardiovascular event-free survival curves after carotid endarterectomy stratified by tertiles of *PDK1–4* expression; *n* = 70. (*D*) Proteomic analysis of different PDK isoenzymes in atherosclerotic plaques vs. adjacent tissue (*n* = 18/group) from BiKE. Data are expressed as mean ± SEM of normalized tandem mass tag (TMT) ratios. Two-tail paired Wilcoxon test was performed for statistical analysis. (*E*) Immunoblot analysis from human carotid plaques showing PDH phosphorylation sites in symptomatic (S) and asymptomatic (AS) patients from BiKE (*n* = 6 samples/group); Mann–Whitney *U* test analysis. (*F*) Quantification of cytokine production in cells isolated from human atherosclerotic plaques (pooled data from 3 independent experiments in triplicate); Kruskal–Wallis ANOVA with Dunn’s post-test analysis. *P < 0.05, **P < 0.01, ***P < 0.001, and ****P < 0.0001.

We examined the expression profile of PDK isoenzymes in human carotid endarterectomy specimens of carotid artery disease patients. RNA-seq analysis revealed that *PDK1* and *PDK4* transcript levels correlate with the levels of several signature pro-inflammatory and destabilizing genes, including macrophage and T-cell-associated genes. Conversely, *PDK2* expression and *PDK3* expression display an inverse gene expression pattern correlating with anti-inflammatory and plaque stabilizing genes, in particular smooth muscle cell marker genes (*Figure.1A*). Remarkably, *PDK1* and *PDK4* gene expression positively correlated with the index of plaque vulnerability (*Figure 1B*). Gene set enrichment analysis (GSEA) of RNA-seq data revealed that genes positively correlating with *PDK1* or *PDK4*, and negatively correlating with *PDK2* or *PDK3*, were enriched in immune-related pathways, e.g. ‘inflammatory_response’, ‘allograft_rejection’, ‘complement’, ‘interferon_gamma_response’, and ‘il6_jak_stat3_signaling’ (see Supplementary material online, *FigureS1*). In contrast, genes that negatively correlated with *PDK1* or *PDK4*, and positively correlated with *PDK2* and *PDK3*, were enriched in pathways especially involved in muscle differentiation (myogenesis) and ‘epithelial_mesenchymal_transition’ (see Supplementary material online, *Figure S1*). Evaluation of PDK isoforms’ expression in plaques from patients with asymptomatic and symptomatic carotid disease revealed a trend towards higher *PDK4* expression and a significant lower *PDK2* expression in plaques from symptomatic patients; no significant differences between groups were seen for *PDK1* and *PDK3* expression (see Supplementary material online, *Figure S2*).

As plaque *PDK1* and *PDK4* transcript levels were associated with inflammation and vulnerability, we next tested whether the expression of these genes could predict future CV events in the same cohort. During the follow-up period (up to 85 months), a total of 17 events were registered from the 70 patients followed up. When dividing the subjects based on PDK mRNA expression for each isoform, CV event-free survival was substantially lower in patients in the highest *PDK1* expression tertile compared with the lowest and middle tertiles (*P* = 0.024; *Figure 1C*). No significant differences were observed for the other PDK isoforms (*Figure 1C*).

Next, we examined the proteomic data from a second biobank (BiKE database) to evaluate whether PDK protein isoforms are differentially expressed in human atherosclerotic plaques compared with non-diseased tissue. This analysis revealed PDK1 protein as the only upregulated isoform in atherosclerotic plaques compared with adjacent, non-diseased, artery tissue (*Figure 1D*); of note, the PDK4 protein isoform was not identified in this specific proteomic analysis (*Figure 1D*).

It is known that PDK over-expression and activity lead to the phosphorylation of PDH, limiting pyruvate metabolism via the TCA cycle.^19^ Western blot analysis of protein extracts from the carotid plaque of symptomatic and asymptomatic patients from BiKE revealed an increase in pSer232-PDH, the specific site for PDK1 phosphorylation (*Figure 1E*). No significant difference for PDH’s common PDK phosphorylation site, pSer293, was observed (*Figure 1E*). Interestingly, we found that the pan-PDK inhibitor DCA inhibits the secretion of IL-6 and IL-8 by *ex vivo*-cultured freshly isolated human atheroma cells in a dose-dependent manner (*Figure 1F*).

**Figure 2:**
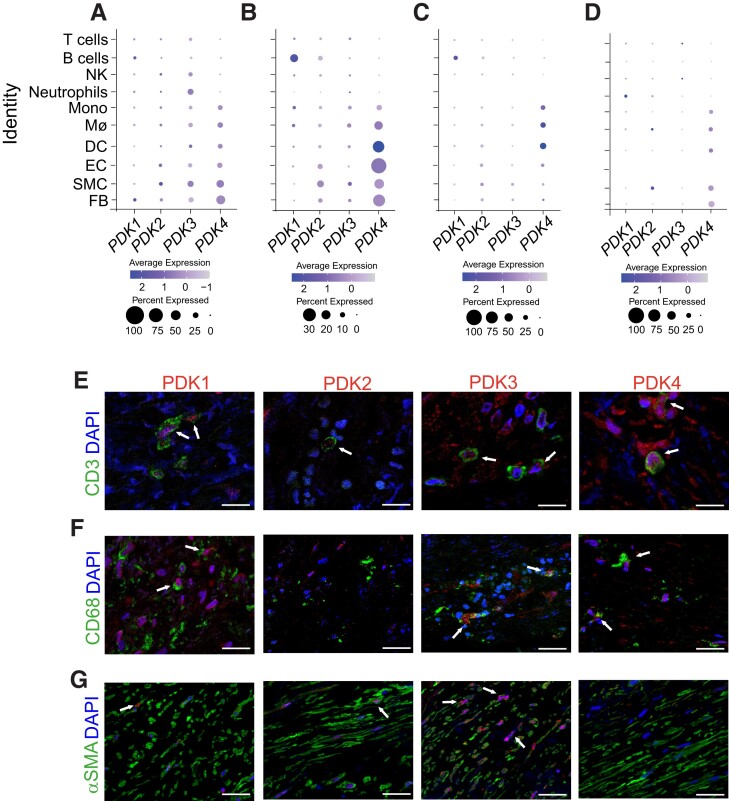
The expression pattern of PDK isoenzymes in human atherosclerotic plaques. (*A*–*D*) Dot plots depicting mRNA levels of PDK isoenzymes in common immune and vascular cell populations defined by their cell-type marker genes in human plaques (public data sets analysed using PlaqView; www.plaqview.com). Dot size depicts the percentage fraction of cells expressing a gene, and dot colour intensity depicts the degree of expression of each gene. (*A*) Data from carotid plaques, *n* = 18^20^; (*B*) data from carotid plaques, *n* = 3^23^; (*C*) data from carotid plaques, *n* = 3^21^; (*D*) data from coronary plaques, *n* = 35.^22^ (*E*, *F*) Plaque material from human carotid endarterectomies was sectioned and representative samples were stained by immunofluorescence to detect PDK1–4 isoenzymes (columns), which are shown in red. Immunofluorescence costaining was performed for the markers for (*E*) CD3^+^ T-cells, (*F*) CD68^+^ macrophages, and (*G*) alpha-smooth muscle cell actin (αSMA), which are shown in green. Slides were stained with the nuclear counterstain DAPI. Pictures were acquired with 40 times magnification. Scale bar = 30 μm. NK, NK cells; Mono, monocytes; Mφ, macrophages; DC, dendritic cells; EC, endothelial cells; SMC, smooth muscle cells; FB, fibroblasts.

In order to identify the expression pattern of PDK isoenzymes within cells in human plaques, first, we accessed and analysed four public data sets of single-cell RNA-seq of atherosclerotic plaques,^20–23^ using the online tool Plaqview (www.plaqview.com).^24^ Overall, this analysis suggested a ubiquitous expression pattern of the different isoforms among the most common immune and vascular cells isolated from human plaque (*Figure 2A–D*) and suggested that in some plaques, *PDK4* can be upregulated in myeloid and vascular cells (*Figure 2B*). Although seemingly with less frequency, data suggested that *PDK1*, *PDK2*, and *PDK3* expression may also be high among these cells (*Figure 2A–D*). Through the immunofluorescence staining of carotid plaques from BiKE, we confirm that all PDK isoforms can be found in CD3^+^ T-cells, while PDK1, PDK3, and PDK4 were the most frequently found isoforms in CD68^+^ macrophages (*Figure 2E* and *F*). Interestingly, αSMA^+^ smooth muscle cells quite often expressed PDK3, but to a lesser extent PDK1 and PDK2, and seemed almost devoid of PDK4 expression in the set of samples analysed (*Figure 2G*).

### 3.2 Inhibition of PDK with DCA inhibits vascular inflammation and atherosclerosis

**Figure 3:**
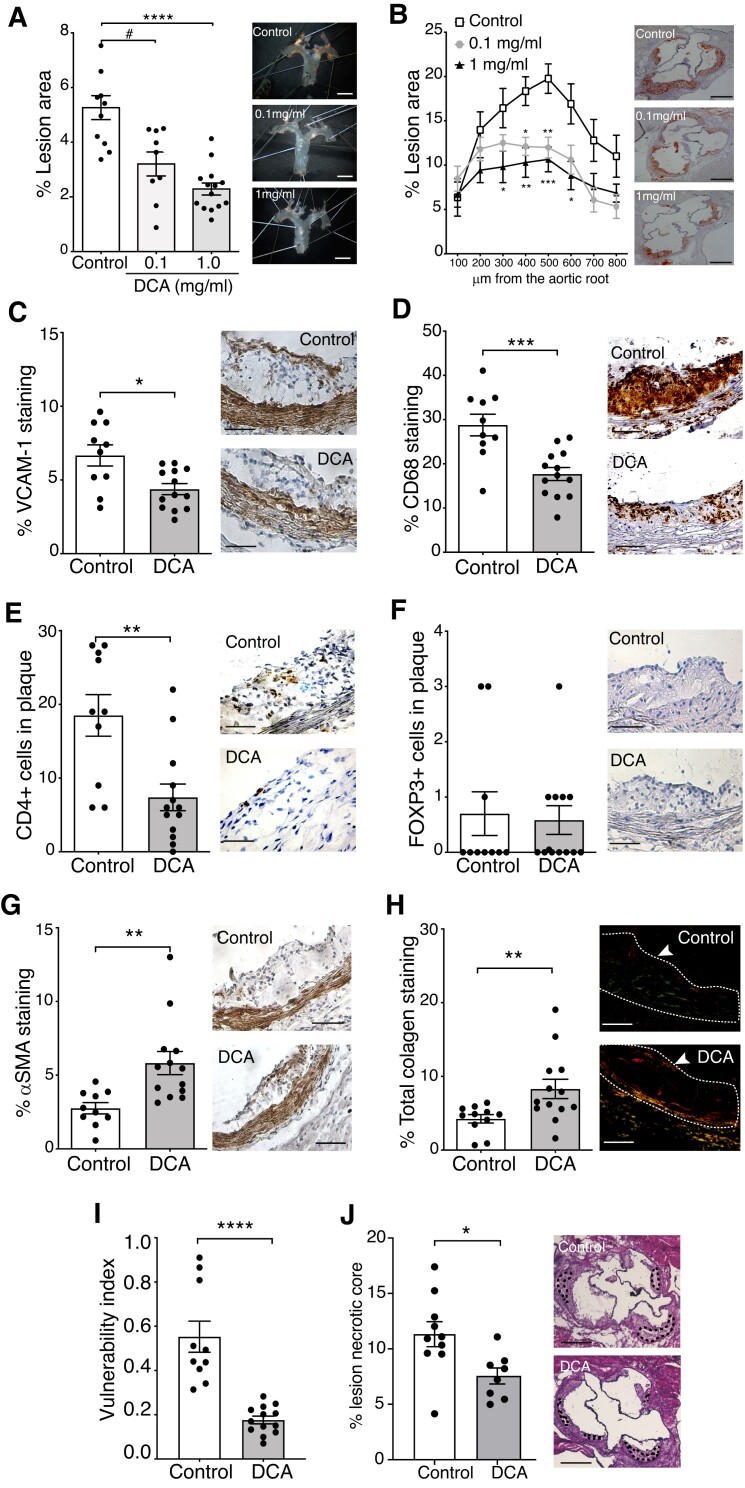
Targeting PDK reduces atherosclerosis and vascular inflammation and increases plaque stability features. (*A*) Percentage lesion areas of Sudan IV *en face* stained aortas. The right panels are representative samples from the control and DCA-treated groups (0.1 and 1 mg/mL; ∼17 and 170 mg/kg/day). (*B*) The per cent lesion area in oil-red-O-stained cross-sections of the aortic root for each group. Right panels show representative micrographs from each group; (*A*, *B*) *n* = 10, 9, and 14 for control, DCA 0.1 mg/mL (∼17 mg/kg/day), and 1.0 mg/mL (∼170 mg/kg/day), respectively; Kruskal–Wallis ANOVA with Dunn’s post-test analysis. Immunohistochemical analysis of (*C*) VCAM-1 and (*D*) CD68− staining and (*E*) CD4− and (*F*) Foxp3-positive cells and (*G*) αSMA immunostaining in the aortic root of controls and DCA-treated mice. (*H*) Collagen evidenced by sirius red staining of controls and DCA-treated mice. Right panels show representative pictures for each group. Original magnification, 40×. Scale bar = 100 μm. (*I*) Vulnerability index as defined in methods. (*J*) Percentage of necrotic core area. Right panels show representative pictures for each group. Original magnification, 40×. Scale bar = 100 μm. All results show the mean ± SEM and are pooled data from 2 independent experiments. (*C–E*, *F–I*) *n* = 10 and 13 for controls and 1 mg/mL for the DCA-treated group, respectively; (*F*) *n* = 10 and 12 for controls and the treated group, respectively; (*J*) *n* = 10 and 8 for controls and the DCA-treated group, respectively. (*C–J*) 1 mg/mL (∼170 mg/kg/day) DCA, Mann–Whitney *U* test analysis. ^#^*P* = 0.05, *P < 0.05, **P < 0.01, ***P < 0.001, and ****P < 0.0001.

The sequences of the four PDK isoenzymes are evolutionary highly conserved: 91.5, 97.5, 95.2, and 92.5% homology are seen between human and mouse PDK1, PDK2, PDK3, and PDK4 protein sequences, respectively (data from Uniprot Alignment tool; https://www.uniprot.org/align). Two different doses of DCA were orally administered to western diet-fed *Apoe−/−* mice, for 8 weeks, to evaluate the effect of PDK inhibition in atherosclerosis. Treatment with this non-species-specific pan-PDK inhibitor substantially impacted plaque formation in a dose-dependent manner, reducing the lesion area by 60% in the aortic arch and by 47% in the aortic root at the highest dose (*Figure 3A* and *B*). No significant differences in body weight were found between groups (see Supplementary material online, *Table S1*). Of note, significantly lower levels of circulating cholesterol but not triglycerides were observed at the endpoint in the high-DCA-dose group; despite trends, no significant difference in lipid levels was observed between groups at earlier timepoints (see Supplementary material online, *Table S1*). A trend towards reduced hepatic cholesterol levels was also observed upon DCA treatment (see Supplementary material online, *Figure S3A*). Further analyses of liver samples from these mice revealed, in general, no pronounced effects of DCA on the expression of lipoprotein metabolism-related genes, except for a trend towards increased *Ldlr* mRNA levels in DCA-treated mice (see Supplementary material online, *Figure S3B*). However, no correlation between *Ldlr* mRNA and plasma cholesterol and triglyceride levels was observed in our mice (see Supplementary material online, *Fig.3C* and *D*).

Because 1 mg/mL DCA treatment (∼170 mg/kg/day) showed more potent atheroprotective effects, this dose was chosen for further mechanistic investigations. Immunohistochemical staining of aortic roots revealed that DCA treatment downregulated VCAM-1 expression (*Figure 3C*) and the infiltration of CD68^+^ macrophages and CD4^+^ T-cells into the plaque (*Figure 3D* and *E*). Although more frequent in plaques from DCA-treated mice, no significant difference in Foxp3^+^ Treg numbers was observed between groups (*Figure 2F*). Notably, the reduction in vascular inflammation was paralleled by the increased content of αSMA^+^ cells (*Figure 3G*) and total collagen levels (*Figure 3H*). Calculation of a vulnerability index that integrates histopathological readouts showed that DCA-treated mice exhibited substantially lower vulnerability indexes than control mice (*Figure 3I*), suggesting that manipulation of the PDK/PDH axis can promote plaque stabilization. Additionally, plaques from DCA-treated mice presented significantly smaller necrotic cores compared with controls (*Figure 3J*).

### 3.3 Inhibition of PDK shifts the immune system in the aorta and spleen

**Figure 4:**
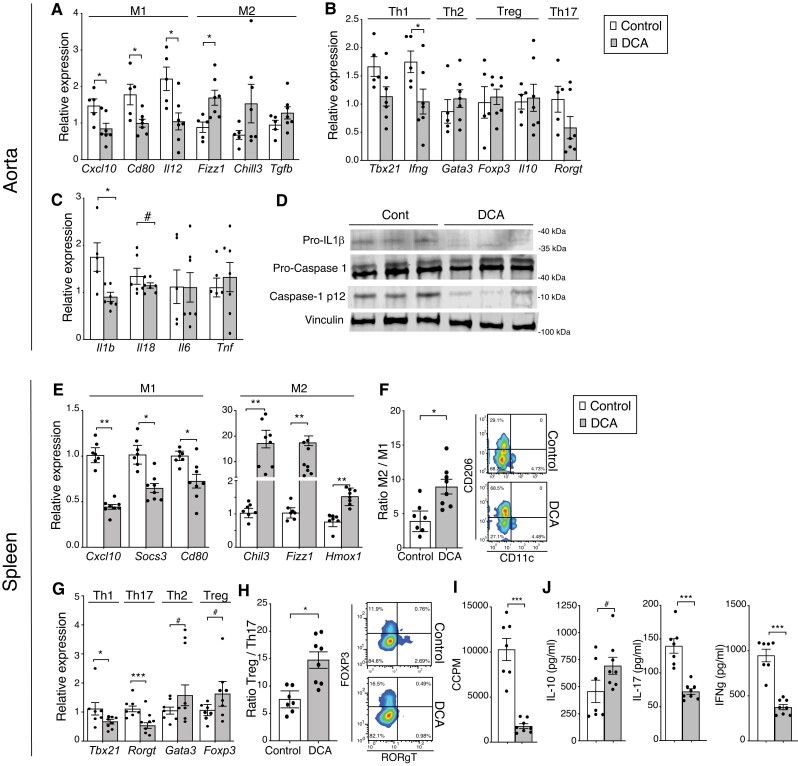
Inhibition of PDK shifts immune response and proliferation. Aortic mRNA quantification of (*A*) M1 and M2 macrophage signature markers, (*B*) CD4 T-cell signature markers and (*C*) aortic mRNA quantification of innate-immunity cytokines in controls and DCA-treated mice; (*A–C*) *n* = 5 and 7 for controls and DCA-treated groups, respectively; Mann–Whitney *U* test analysis. (*D*) Expression of the NLRP3 inflammasome-related proteins IL-1β, pro-caspase 1, and cleaved caspase 1 and the loading control vinculin in aortas from controls and DCA-treated mice (*n* = 3 samples/group). (*E*) mRNA quantification of M1 and M2 macrophage signature genes in the spleen (*n* = 7 and 8 for control and DCA-treated groups, respectively); Mann–Whitney *U* test analysis. (*F*) M2/M1 ratio in the spleen of controls and DCA-treated mice (*n* = 7 and 8 for control and DCA-treated groups, respectively); Mann–Whitney *U* test analysis. Right panels show representative plots for M2 (CD206+) and M1 (CD11c+) macrophages within gated CD11b+ F4/80+ splenocytes. (*G*) mRNA quantification of CD4+ T-cell signature markers in the spleen. (*H*) Treg/Th17 ratio. Right panels show representative flow cytometry plots for Treg (Foxp3^+^) and Th17 (RorγT^+^) T-cells from the spleen of controls and DCA-treated mice. Representative plots show percentages among CD4^+^ T-cells. (*I*) Quantification of splenocyte proliferation in vitro from controls and DCA-treated mice, in response to anti-CD3 and anti-CD28 stimulation for 48 h. (*J*) Cytokines in supernatants from the proliferation assay; (*G–J*) *n* = 7 and 8 for control and DCA-treated groups, respectively; Mann–Whitney *U* test analysis. (*A–J*) 1 mg/mL (∼170 mg/kg/day) DCA. The results show the mean ± SEM and are representative of two independent experiments. #*P* = 0.05, *P < 0.05, **P < 0.01, ***P < 0.001, and ****P < 0.0001.

Whether the effects of DCA treatment influence innate and adaptive immunity was investigated in the aorta and spleen of DCA-treated and control mice. Analysis of aortic mRNA indicated a major shift from M1 towards M2 macrophage polarization in DCA-treated mice (*Figure 4A*). T-cell marker transcripts were less skewed in the aorta, and only *Ifng* mRNA was significantly reduced in the DCA-treated group compared with controls (*Figure 4B*). In line with the plaque immunostaining data, arterial *Foxp3* expression was not significantly affected by DCA treatment (*Figure 4B*). Further analysis of transcripts of innate immune cytokines showed that the expression of the NLRP3 inflammasome-dependent cytokines *Il1b* and *Il18* was also reduced upon DCA treatment (*Figure 4C*). Thus, western blot analyses of aortic protein extracts showed that pro-IL1β was downregulated in samples from DCA-treated mice compared with controls (*Figure 4D*). Further analysis showing that cleaved caspase 1 was also reduced upon DCA treatment (*Figure 4D*) indicate that PDK inhibition also halted the activation step of NLRP3 inflammasome in the vascular wall.

DCA-treated mice presented decreased pro-inflammatory and increased anti-inflammatory responses in the spleen, including reduced mRNA levels of M1 markers and increased mRNA levels of M2 markers (*Figure 4E*) and an increased ratio of M2/M1 macrophages (*Figure 4F*) detected by flow cytometry. We observed significantly reduced levels of T-cell pro-inflammatory markers, namely, the Th1 marker *Tbx21* and the Th17 marker *Rorγ*t, and a trend towards increased levels of T-cell anti-inflammatory markers, namely, the Treg marker *Foxp3* and the Th2 marker *Gata3* (*Figure 4G*). Using flow cytometry, we observed that the Treg/Th17 ratio was increased in the spleen of DCA-treated mice compared with controls (*Figure 4H*). *In vitro*, T-cells from DCA-treated mice presented a reduced capacity to proliferate in response to anti-CD3/anti-CD28 stimulation (*Figure 4I*) and showed a skewed anti-inflammatory response with increased production of IL-10 and decreased production of IFNγ and IL-17A (*Figure 4J*). No significant differences in blood cell counts were observed between groups (see Supplementary material online, *Figure3*). In the liver, significantly lower levels of *Cd4* and *Rorc* (encoding *RORγt*) mRNA were observed upon DCA treatment; no significant differences for M1–M2 markers or other T helper cell markers were observed between groups (see Supplementary material online, *Figure4*).

### 3.4 DCA-mediated rescue of PDH activity in the artery wall influences succinate secretion and the release of IL-1β by macrophages

**Figure 5:**
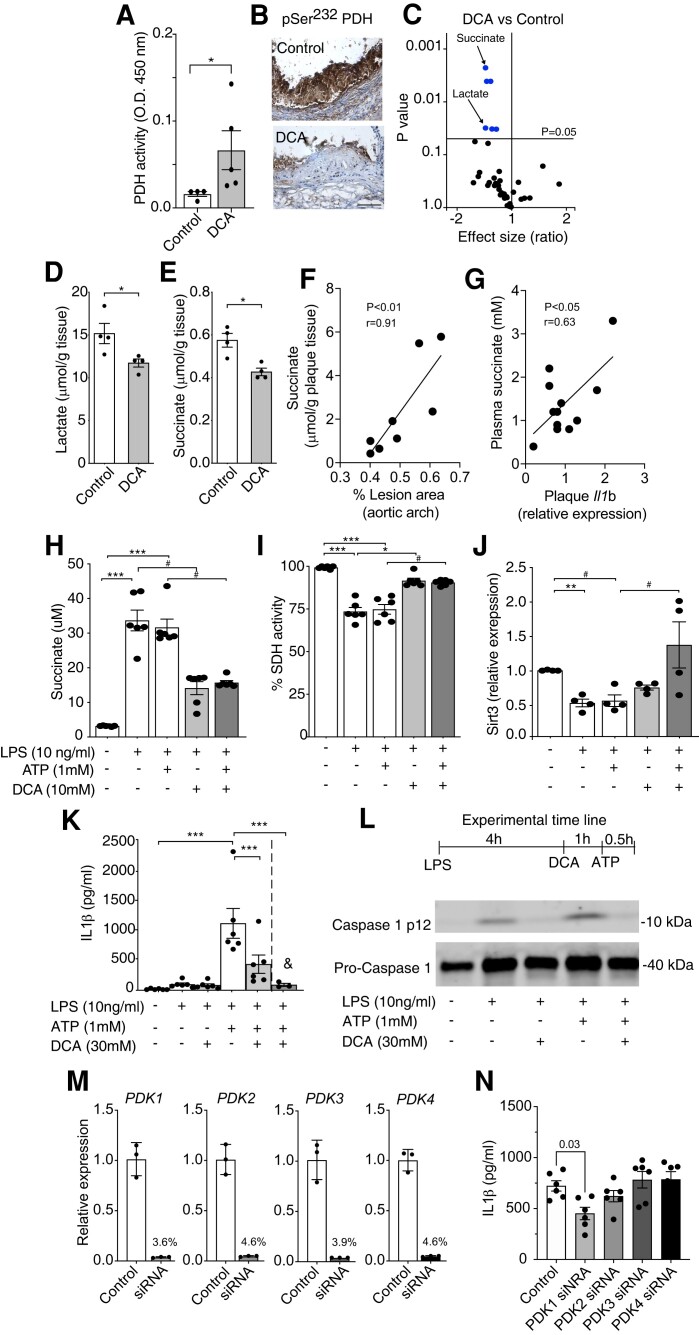
Inhibition of PDK reduces the pro-inflammatory response of the metabolite succinate in atherosclerosis. (*A*) Enzymatic PDH activity from protein extract of aortic tissue from controls and DCA-treated mice (*n* = 4 and 5, respectively); Mann–Whitney *U* test analysis. (*B*) Representative immunostainings of phosphorylated PDH E1a (serine 232) in aortic roots from control and DCA-treated mice. (*C*) 1H-NMR metabolomic analysis of aortic tissue from controls and DCA-treated mice (*n* = 4/group); Mann–Whitney *U* test analysis. Effect size differences and *P*-value between groups are shown. Quantification of (*D*) lactate and (*E*) succinate in the aortic tissue of controls and DCA-treated mice (*n* = 4/group); Mann–Whitney *U* test analysis. (*A–G*) 1 mg/mL (∼170 mg/kg/day) DCA. (*F*) Correlation between aortic levels of succinate (1H-NMR) and % of plaque in the aortic arch of controls and DCA-treated mice (*n* = 8); linear regression analysis. (*G*) Correlation between plasma levels of succinate and aortic mRNA levels of *Il1b* in controls and DCA-treated mice (*n* = 11); linear regression analysis. Effect of DCA on (*H*) succinate release (*I*) SDH activity (*n* = differentiated cells from six individual mice, treated in duplicates); Kruskal–Wallis ANOVA with Dunn’s post-test analysis. (*J*) *Sirt3* mRNA levels (*n* = differentiated cells from four individual mice, treated in duplicates); Kruskal–Wallis ANOVA with Dunn’s post-test analysis. (*K*) IL-1β release by BMDMs upon NLRP3 priming and activation, with DCA added before and (^&^) after LPS stimulation (*n* = differentiated cells from six individual mice, treated in duplicates); Kruskal–Wallis ANOVA with Dunn’s post-test analysis. (*L*) Upper panel shows the experimental timeline for NLRP3 inflammasome activation and DCA treatment. Bottom panels, analysis of the protein expression of pro-caspase 1 and cleaved caspase 1 in representative BMDM protein extracts. (*M*) Evaluation of transfection efficiency of THP-1 cells incubated with small interfering RNA against PDK1–4 (PDK1–4 siRNA) or scrambled negative siRNA control (control), as described in methods (*n* = 3, pooled data from two experiments, in duplicate wells); the percentage expression after siRNA transfection is shown on the top of the bars. (*N*) THP-1 cells transfected with PDK1–4 siRNA or scrambled control siRNA and differentiated with PMA (10 nM for 24 h) were stimulated with LPS and ATP for NLRP3 priming and activation and of IL-1β release (*n* = 6, pooled data from two experiments, in triplicate wells). #*P* < 0.07, *P < 0.05, **P < 0.01, and ***P < 0.001.

DCA inhibits PDK activity, thereby allowing PDH to be in its unphosphorylated active form and promoting pyruvate metabolism via the TCA cycle.^19^ We found that aortic protein extracts from DCA-treated mice presented significantly increased PDH activity (*Figure 5A*). This mirrored the immunohistochemistry analyses of aortas showing reduced pSer^232^-PDH upon DCA treatment (*Figure 5B*). NMR-based metabolomics of the mouse aortas revealed that DCA treatment reduced vascular levels of lactate, as well as betaine, but most significantly succinate (*Figure 5C–E*; and Supplementary material online, *Table S2*). Significant reductions in the branched-chain amino acids leucine and isoleucine were also observed in the aortas of DCA-treated mice (see Supplementary material online, *Table S2*). Interestingly, succinate levels in the plaques of DCA-treated mice and controls correlated significantly with plaque area in the aortic arch (*Figure 5F*), while plasma succinate levels correlated significantly with aortic *Il1b* mRNA levels (*Figure 5G*) in the same mice. A trend towards *Pdk1* and *Pdk4* mRNA levels correlating with CD68 staining content in the plaques was also observed (see Supplementary material online, *Figure 3*); no correlation between plasma succinate levels and PDK isoforms’ mRNA expression in the plaque was seen (data not shown).

Cultured murine macrophages were used to investigate whether PDK inhibition and PDHc activity stimulation can directly modulate succinate release by macrophages *in vitro*. LPS stimulation increased the production of succinate, which could be significantly inhibited by DCA (*Figure 5H*). These data are in line with the finding that DCA significantly rescued the activity of the succinate degrading enzyme succinate dehydrogenase (SDH), which is inhibited upon LPS stimulation (*Figure 5I*). Analysis of the expression of sirtuin-3 (*Sirt3*), a known activator of SDH activity, showed that DCA can rescue the expression of *Sirt3*, which is also reduced by LPS in macrophages (*Figure 5J*).

Considering the parallel effects on succinate levels, inflammasome activation, and IL-1β production in the aorta of DCA-treated mice, we evaluated whether the inflammasome is affected by DCA in macrophage cultures. Treatment with DCA before LPS stimulation, a condition that reduces succinate production, significantly reduced IL-1β release (*Figure 5K*). Interestingly, DCA treatment shortly before ATP stimulation showed an even more pronounced effect on the inhibition of IL-1β (*Figure 5K*). Corroborating these findings, we observed decreased expression of cleaved caspase 1 in protein extracts from macrophages treated with DCA before LPS or before ATP (*Figure 5L*). No effects of ATP were seen in the modulation of succinate secretion (*Figure 5K* and *L*), and no differences in pro-caspase 1 protein levels were seen induced by DCA treatment (*Figure 5L*). Of note, DCA treatment did not substantially influence mitochondrial LPS-induced reactive oxygen species (ROS) production by BMDMs, and no signs of toxicity to the macrophage cultures were seen for DCA in our experiments (see Supplementary material online, *Figure S6A and B*).

Because DCA is a pan-inhibitor, in our earlier experiments, it was not possible to pinpoint whether any of the PDK isoforms play a hierarchical higher role in the regulation of IL-1β secretion by macrophages *in vivo*. Nevertheless, through successfully silencing mRNA encoding the four PDK isoforms in THP-1 macrophage cultures (*Figure 5M*), we found that only PDK1 siRNA significantly reduced IL-1β secretion compared with control scrambled siRNA (*Figure 5N*).

#### 3.4.1 DCA impairs succinate-mediated GPR91 signalling and NLRP3 inflammasome activation in macrophages

**Figure 6:**
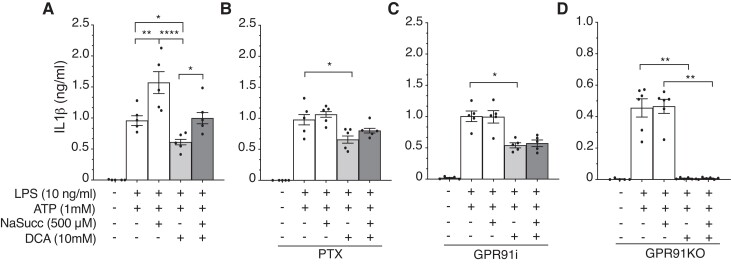
Inhibition of PDK reduces GPR91-mediated inflammasome activation. Quantification of IL-1β released from BMDMs upon NLRP3 inflammasome activation with LPS and ATP and/or pre-treated with DCA 1 h before ATP. (*A*) Effect of treatment with sodium succinate (NaSucc) 1 h prior to NLRP3 inflammasome activation with LPS and ATP and/or pre-treated with DCA. (*B*) Effect of treatment with 200 ng/mL pertussis toxin (PTX) for 2 h and NaSucc for 1 h before NLRP3 inflammasome activation with LPS and ATP and/or pre-treated with DCA. (*C*) Effect of treatment with 50 µM GPR91 inhibitor (GPR91i) for 2 h and NaSucc for 1 h before NLRP3 inflammasome activation with LPS and ATP and/or pre-treated with DCA. (*D*) Effect of treatment with NaSucc 1 h prior to NLRP3 inflammasome activation with LPS and ATP and/or pre-treated with DCA on the release of IL-1β by BMDMs obtained from GPR91KO mice. Results show mean ± SEM of IL-1β production (*n* = differentiated cells from (*A–C*) five and (*D*) six individual mice, treated in duplicate). (*A–D*) Kruskal–Wallis ANOVA with Dunn’s post-test analysis. *P < 0.05, **P < 0.01, ***P < 0.001, and ****P < 0.0001.

Although mostly seen as an intermediate metabolite in the TCA cycle, it has been shown that succinate regulates IL-1β gene expression through the stabilization of hypoxia-inducible factor 1 alpha (HIF-1α).^25^ Moreover, succinate acting as an extracellular signal through the Gi-coupled GPR91 has been demonstrated to synergize with Toll-like receptors and boost immune responses.^26^ We found that the exogenous addition of sodium succinate significantly increased IL-1β and counteracted the DCA-mediated inhibition of IL-1β secretion by macrophages (*Figure 6A*). A similar experimental design using diethyl succinate, a more membrane-permeable succinate analogue and inactive to GPR91, failed to substantially increase IL-1β production or to rescue the DCA-mediated inhibition of IL-1β secretion (see Supplementary material online, *Figure S7A*), suggesting that the extracellular conceivably GPR91-mediated effects of succinate could play a major role in our model system. Corroborating with the latter, we observed that the pre-treatment of macrophages with pertussis toxin, which blocks G-protein-coupled receptor signalling, and coincubation with a specific GPR91 antagonist both prevented succinate-driven effects on IL-1β (*Figure 6B* and *C*). Importantly, the effects of sodium succinate on NLRP3 inflammasome were eliminated in macrophages from GPR91KO mice, in which DCA treatment led to complete inhibition of IL-1β secretion (*Figure 6D*). Notably, IL-1β secretion by macrophages from GPR91KO mice was approximately half of that observed in macrophages from wild-type littermate controls (*Figure 6*, and see Supplementary material online, *Figure 7*).

## 4. Discussion

By suppressing the activity of PDHc, PDK isoenzymes are at the crossroad of glycolysis and glucose oxidation, regulating the balance between pro- and anti-inflammatory responses.^27^ We have demonstrated for the first time that the overexpression of *PDK1* and *PDK4* is associated with vascular inflammation in human plaques and that *PDK1* expression is associated with a more unstable plaque phenotype and could predict secondary CV events. Moreover, we demonstrate that targeting PDKs and promoting PDHc-dependent metabolism with DCA skews the immune system, inhibits vascular inflammation and atherogenesis, and promotes plaque stability features in *Apoe*−/− mice.

PDK exists in four isoforms that display unique tissue distribution and enzymatic activities, suggesting a different role depending on the cell type.^28^ Abnormal PDK activity has been previously associated with diabetes, metabolic disorders, cardiomyopathy, and several cancers.^8^ We have found that in human atherosclerosis, *PDK1* and *PDK4* expression positively correlates with pro-inflammatory markers related to CD4^+^ T-cells and macrophage activation. This finding goes in line with studies showing that *PDK1* is overexpressed during chronic inflammation, where highly glycolytic and proliferative cells may predominate, e.g. rheumatoid arthritis and multiple sclerosis.^17,29^

Both the innate and adaptive immune responses orchestrate all stages of atherosclerosis. Type 1 immune responses, including the secretion of pro-inflammatory cytokines such as TNF, IL-1β, and IFNγ by M1 macrophages and Th1 cells, have been associated with the acceleration of disease and the destabilization of plaques. Conversely, there is strong evidence indicating that promoting the type 2-mediated responses of M2 macrophages and Tregs can reduce disease burden. Previous characterization of the PDK isoforms in immune cells indicated that PDK1, PDK2, and PDK4 would be involved in M1 macrophage polarization and their pro-inflammatory responses.^27,30,31^ PDK1 expression can be upregulated upon TCR stimulation in CD4^+^ T-cells^32^ and inhibition of PDK1 by DCA protects mice from developing experimental autoimmune encephalitis, by limiting the response of Th17 cells.^18^

Among all PDK isoenzymes, the expression of *PDK4* has been implicated in energy homeostasis and rapidly changes under different metabolic conditions. Hence, it has been proposed that PDK4 plays an important role in the metabolic process that contribute to the osteogenic switch in SMCs during the development of vascular calcification.^33^ In our study, *PDK1* expression and *PDK4* expression were associated with the upregulation of inflammatory pathways in carotid plaques, and a trend towards increased *PDK4* was seen in plaques from patients with symptomatic unstable atherosclerosis disease. Considering that plaques from these patients should mostly present increased inflammation and impaired SMC responses,^34^ we speculate that both immune and vascular cells could be affected by the altered expression of these enzymes. In line with this thought, we provide data supporting the ubiquitous expression of PDK isoenzymes among cells in human plaques. Thus, corroborating to the possibility that PDK inhibition can also affect vascular cells, it has been shown that DCA influences SMC apoptosis and prevent their dedifferentiation, driving reduced SMC proliferation and neointima formation in animal models of restenosis, including a humanized balloon injury model with human internal mammary arteries implanted to T-cell-deficient Rowett nude rats.^35^

Whether PDK inhibition can influence human atherosclerosis has never been tested. In our preclinical setting, blocking PDKs with DCA led to profound effects on the immune system that reduced atherosclerosis and promoted a fibrous cap-related response of SMCs in the artery wall. DCA is known to inhibit all PDK isoforms at the mM range *in vitro*, with potency estimated to be higher against PDK2 < PDK 1 ≃ PDK4 < PDK3,^36^ and it has been shown that DCA treatment diminishes the phosphorylation of the three serine phosphorylation sites on PDH (PDH_S232_, PDH_S293_, and PDH_S300_) in different cancer cell lines. Interestingly, the latter study has shown a higher inhibitory effect of DCA against the phosphorylation of PDH_S232_, which is the specific site of PDK1 phosphorylation, in A375 and MeWo cells.^37^ This knowledge corroborates with our human data and reinforces the notion that metabolic reprograming of the PDK/PDH axis, potentially through inhibition of PDK1, could be a relevant strategy to prevent and treat CVD in humans. While isoform-specific PDK inhibitors are not ready for clinical trials, it should be mentioned that DCA has shown promising results clinically for the treatment of pulmonary hypertension (ClinicalTrials.gov; NCT01083524)^38^ and is currently under investigation for the treatment of endometriosis (ClinicalTrials.gov; NCT0404608),^39^ indicating that its efficacy towards atherosclerotic CVDs could potentially be evaluated in the near future. Besides, the fact that DCA can influence endothelial function, stabilize haemodynamics, and reverse microvascular endotoxin tolerance, in the context of experimental sepsis,^40^ suggests that other beneficial CV effects may be achieved with the restoration of PDHc activity in the context of vascular inflammation.^41^

A major outcome of inhibiting PDK activity, and promoting a healthy mitochondrial PDHc metabolism, inhibited especially innate immunity, seen by the downregulation of M1-macrophage markers, smaller necrotic cores, and decreasing NLRP3 inflammasome and the secretion of IL-1β in the aorta. Considering the positive results of the Canakinumab Anti-inflammatory Thrombosis Outcome Study (CANTOS) showing that IL-1β blockade significantly reduced the number of CV events,^42^ as well as CV mortality in patients that responded with reduced hsCRP,^43,44^ DCA and/or other PDK blockers emerge as an attractive alternative to inhibit the deleterious effects of this pathway on CVD. Notably, canakinumab treatment significantly increased the risk of fatal infections,^42^ which has not been evident in clinical trials using the PDK inhibitor DCA.

A major characteristic of M1-polarized macrophages is a truncated TCA cycle, which leads to the intracellular accumulation and secretion of succinate.^45^ Either intracellularly, stabilizing HIF1a, or extracellularly, interacting with the metabolite sensing GPR91, succinate has been implicated in the regulation of IL-1β production.^25,46^ Metabolomic analysis of mouse aortas revealed that DCA treatment most significantly reduced vascular succinate levels, which coincided with the reduced M1 responses and NLRP3 inflammasome activation found in the same group. Although to which extent intracellular and extracellular succinates influence the overall pool of IL-1β produced by activated macrophages remains unclear, our data suggest that succinate/GPR91 signalling in the artery wall is an important feed-forward loop promoting vascular inflammation. Importantly, our *in vitro* and *in vivo* data propose that targeting PDK can be used to disrupt this loop.

Succinate accumulation occurs most often due to impaired SDH activity, e.g. patients carrying mutations in the SDH complex genes display elevated levels of plasma succinate.^47^ SIRT3 is a mitochondrial NAD+-dependent histone deacetylase pinpointed as a critical regulator of SDH.^48^ Interestingly, it has been suggested that SIRT3-dependent deacetylation, which activates SDH,^49^ can also regulate GPR91 expression in the context of non-alcoholic fatty liver disease (NAFLD).^50^ The fact that DCA treatment restored *Sirt3* expression and SDH activity in LPS-stimulated macrophages revealed an unknown link with the PDK/PDH axis. Whether NAD+ and niacinamide would be involved in DCA-mediated succinate production will require further investigation. Succinate production in activated macrophages can be driven in parallel by increased glutamine-dependent anaplerosis and the ‘GABA-shunt’ pathway;^25^ thus, to which extent the PDK/PDH axis regulates the GABA-shunt pathway also warrants further research.

Because DCA was administered orally, it is likely that PDK inhibition could lead to other consequences than just affecting the immune system. Many years ago, orally given DCA, at high doses, was considered a potential lipid-lowering therapy in humans, which unfortunately induced neurological side effects that halted further development for this clinical application^51,52^; it is now known that the latter adverse effect of DCA, a reversible sensory and motor peripheral neuropathy, is influenced by age and is often found in individuals carrying a specific genetic haplotype of the gene encoding the enzyme glutathione transferase zeta 1 (*GSTZ1*), which is involved in the biotransformation of DCA and influencing the generation of toxic molecules.^36,53^

We show that long-term exposure to a high DCA dose could significantly reduce cholesterol levels within the last two weeks of treatment in Apoe-/- mice. Interestingly, we observed a trend towards increased hepatic *Ldlr* expression in DCA-treated mice, which has previously been suggested^54^ and in a long-term therapeutic perspective could further help decelerate atherosclerosis progression. However, the fact that hepatic *Ldlr* mRNA levels did not correlate with plasma lipids, as well as the finding that DCA-treated mice presented reduced hepatic cholesterol levels, suggests that increased receptor-mediated clearance may not be the only mechanism regulating cholesterol metabolism upon high doses of DCA. In part due to the limitations of our study design, which did not take into consideration gene regulation at different feeding states, and only one timepoint was investigated, the full spectrum of mechanisms triggered by DCA on lipoprotein metabolism remains uncertain. Nevertheless, the fact that a 10-fold lower dose of DCA led to similar atheroprotective effects as the highest dose, without influencing plasma lipids, suggests that this mechanism was secondary to influencing atherogenesis. Future investigations will be needed to fully understand the potential effects of modulating the PDK/PDH axis on lipid metabolism, including synthesis, clearance, and excretion, as well as if such effects are target specific considering lipid changes were seen only upon a high DCA dose.

The present study has of course other limitations. The observational approach, showing associations between the expression of PDK isoenzymes and symptomatic disease, and the association between high *PDK1* expression and future CV events that was performed in a relatively small cohort of patients do not allow conclusions of causality. Although the pharmacological restoration of arterial PDHc activity in our pre-clinical study would support causality, clinical studies will be necessary to establish the beneficial effects of PDK inhibition in human CVD. In this context, it is interesting that we found succinate—a metabolite that we show could promote IL-1β production in the arterial wall—as a potential surrogate marker of vascular PDHc activity. Although succinate could not be measured in the already processed samples from the biobanks used in the study, it will be interesting to evaluate it in the future, as well as to calculate the prognostic value of plaque or plasma succinate levels for CVD events. Hence, further studies to continue exploring the role of GPR91-mediated signalling in atherosclerosis will be needed.

In conclusion, we identified the PDH/PDK axis as a central metabolic regulator of vascular inflammation, linking glycolysis and mitochondrial PDHc with succinate/GPR91 signalling that can skew the immune system systemically and locally in the artery wall. The observation that PDK inhibition, which restores PDHc activity and reduces succinate/GPR91 pro-inflammatory signalling in the vessel wall, also inhibits the pro-atherogenic and plaque destabilizing pathway of the NLRP3 inflammasome activation and IL-1β secretion emphasizes the potential of metabolic reprograming to prevent and treat CVDs. The fact that *PDK1* expression was significantly associated with IL-1β secretion in our model, and future CV events in humans, proposes that secondary prevention could be a valuable way forward to the use of PDK inhibition clinically. Further research should investigate whether DCA and other more specific and more potent PDK inhibitors have similar effects on the immune system, as well as other disease-modulating factors, such as the response of vascular cells and lipid metabolism.

## Supplementary material

Supplementary material is available at *Cardiovascular Research* online.

## Supplementary Material

cvad038_Supplementary_Data

## Data Availability

The CPIP data sets generated and/or analysed during the current study are not publicly available due the sensitive nature of the data; requests to access the data set from qualified researchers trained in human subject confidentiality protocols may be sent to Isabel Goncalves at Lund University. Other data will be available on request from the corresponding author.

## References

[1] Nichols M , Townsend N, Scarborough P, Rayner M. Cardiovascular disease in Europe 2014: epidemiological update. Eur Heart J 2014;35:2950–2959. 25139896 10.1093/eurheartj/ehu299

[2] Ketelhuth DF , Hansson GK. Modulation of autoimmunity and atherosclerosis—common targets and promising translational approaches against disease. Circ J 2015;79:924–933. 25766275 10.1253/circj.CJ-15-0167

[3] Mills EL , Kelly B, O’Neill LAJ. Mitochondria are the powerhouses of immunity. Nat Immunol 2017;18:488–498. 28418387 10.1038/ni.3704

[4] Palsson-McDermott EM , O’Neill LAJ. Targeting immunometabolism as an anti-inflammatory strategy. Cell Res 2020;30:300–314. 32132672 10.1038/s41422-020-0291-z PMC7118080

[5] Forteza MJ , Ketelhuth DFJ. Metabolism in atherosclerotic plaques: immunoregulatory mechanisms in the arterial wall. Clin Sci (Lond) 2022;136:435–454. 35348183 10.1042/CS20201293 PMC8965849

[6] Joseph P , Tawakol A. Imaging atherosclerosis with positron emission tomography. Eur Heart J 2016;37:2974–2980. 27125951 10.1093/eurheartj/ehw147

[7] Tomas L , Edsfeldt A, Mollet IG, Perisic Matic L, Prehn C, Adamski J, Paulsson-Berne G, Hedin U, Nilsson J, Bengtsson E, Goncalves I, Bjorkbacka H. Altered metabolism distinguishes high-risk from stable carotid atherosclerotic plaques. Eur Heart J 2018;39:2301–2310. 29562241 10.1093/eurheartj/ehy124 PMC6012762

[8] Patel MS , Nemeria NS, Furey W, Jordan F. The pyruvate dehydrogenase complexes: structure-based function and regulation. J Biol Chem 2014;289:16615–16623. 24798336 10.1074/jbc.R114.563148 PMC4059105

[9] Zhang S , Hulver MW, McMillan RP, Cline MA, Gilbert ER. The pivotal role of pyruvate dehydrogenase kinases in metabolic flexibility. Nutr Metab (Lond) 2014;11:10. 24520982 10.1186/1743-7075-11-10 PMC3925357

[10] Bowker-Kinley MM , Davis WI, Wu P, Harris RA, Popov KM. Evidence for existence of tissue-specific regulation of the mammalian pyruvate dehydrogenase complex. Biochem J 1998;329(Pt 1):191–196. 9405293 10.1042/bj3290191 PMC1219031

[11] Sorbi S , Bird ED, Blass JP. Decreased pyruvate dehydrogenase complex activity in Huntington and Alzheimer brain. Ann Neurol 1983;13:72–78. 6219611 10.1002/ana.410130116

[12] McFate T , Mohyeldin A, Lu H, Thakar J, Henriques J, Halim ND, Wu H, Schell MJ, Tsang TM, Teahan O, Zhou S, Califano JA, Jeoung NH, Harris RA, Verma A. Pyruvate dehydrogenase complex activity controls metabolic and malignant phenotype in cancer cells. J Biol Chem 2008;283:22700–22708. 18541534 10.1074/jbc.M801765200 PMC2504897

[13] Edsfeldt A , Goncalves I, Grufman H, Nitulescu M, Duner P, Bengtsson E, Mollet IG, Persson A, Nilsson M, Orho-Melander M, Melander O, Bjorkbacka H, Nilsson J. Impaired fibrous repair: a possible contributor to atherosclerotic plaque vulnerability in patients with type II diabetes. Arterioscler Thromb Vasc Biol 2014;34:2143–2150. 25035341 10.1161/ATVBAHA.114.303414

[14] Perisic L , Aldi S, Sun Y, Folkersen L, Razuvaev A, Roy J, Lengquist M, Akesson S, Wheelock CE, Maegdefessel L, Gabrielsen A, Odeberg J, Hansson GK, Paulsson-Berne G, Hedin U. Gene expression signatures, pathways and networks in carotid atherosclerosis. J Intern Med 2016;279:293–308. 26620734 10.1111/joim.12448

[15] Perisic L , Hedin E, Razuvaev A, Lengquist M, Osterholm C, Folkersen L, Gillgren P, Paulsson-Berne G, Ponten F, Odeberg J, Hedin U. Profiling of atherosclerotic lesions by gene and tissue microarrays reveals PCSK6 as a novel protease in unstable carotid atherosclerosis. Arterioscler Thromb Vasc Biol 2013;33:2432–2443. 23908247 10.1161/ATVBAHA.113.301743

[16] Matic L P , Rykaczewska U, Razuvaev A, Sabater-Lleal M, Lengquist M, Miller CL, Ericsson I, Rohl S, Kronqvist M, Aldi S, Magne J, Paloschi V, Vesterlund M, Li Y, Jin H, Diez MG, Roy J, Baldassarre D, Veglia F, Humphries SE, de Faire U, Tremoli E, Odeberg J, Vukojevic V, Lehtio J, Maegdefessel L, Ehrenborg E, Paulsson-Berne G, Hansson GK, Lindeman JH, Eriksson P, Quertermous T, Hamsten A, Hedin U. Phenotypic modulation of smooth muscle cells in atherosclerosis is associated with downregulation of LMOD1, SYNPO2, PDLIM7, PLN, and SYNM. Arterioscler Thromb Vasc Biol 2016;36:1947–1961. 27470516 10.1161/ATVBAHA.116.307893 PMC8991816

[17] Bian L , Josefsson E, Jonsson IM, Verdrengh M, Ohlsson C, Bokarewa M, Tarkowski A, Magnusson M. Dichloroacetate alleviates development of collagen II-induced arthritis in female DBA/1 mice. Arthritis Res Ther 2009;11:R132. 19723321 10.1186/ar2799 PMC2787291

[18] Gerriets VA , Kishton RJ, Nichols AG, Macintyre AN, Inoue M, Ilkayeva O, Winter PS, Liu X, Priyadharshini B, Slawinska ME, Haeberli L, Huck C, Turka LA, Wood KC, Hale LP, Smith PA, Schneider MA, MacIver NJ, Locasale JW, Newgard CB, Shinohara ML, Rathmell JC. Metabolic programming and PDHK1 control CD4+ T cell subsets and inflammation. J Clin Invest 2015;125:194–207. 25437876 10.1172/JCI76012 PMC4382238

[19] Fouque F , Brivet M, Boutron A, Vequaud C, Marsac C, Zabot MT, Benelli C. Differential effect of DCA treatment on the pyruvate dehydrogenase complex in patients with severe PDHC deficiency. Pediatr Res 2003;53:793–799. 12621116 10.1203/01.PDR.0000057987.46622.64

[20] Slenders L , Landsmeer LPL, Cui K, Depuydt MAC, Verwer M, Mekke J, Timmerman N, van den Dungen NAM, Kuiper J, de Winther MPJ, Prange KHM, Ma WF, Miller CL, Aherrahrou R, Civelek M, de Borst GJ, de Kleijn DPV, Asselbergs FW, den Ruijter HM, Boltjes A, Pasterkamp G, van der Laan SW, Mokry M. Intersecting single-cell transcriptomics and genome-wide association studies identifies crucial cell populations and candidate genes for atherosclerosis. Eur Heart J Open 2022;2:oeab043. 10.1093/ehjopen/oeab043 PMC8841481 35174364

[21] Pan H , Xue C, Auerbach BJ, Fan J, Bashore AC, Cui J, Yang DY, Trignano SB, Liu W, Shi J, Ihuegbu CO, Bush EC, Worley J, Vlahos L, Laise P, Solomon RA, Connolly ES, Califano A, Sims PA, Zhang H, Li M, Reilly MP. Single-cell genomics reveals a novel cell state during smooth muscle cell phenotypic switching and potential therapeutic targets for atherosclerosis in mouse and human. Circulation 2020;142:2060–2075. 32962412 10.1161/CIRCULATIONAHA.120.048378 PMC8104264

[22] Chowdhury RR , D’Addabbo J, Huang X, Veizades S, Sasagawa K, Louis DM, Cheng P, Sokol J, Jensen A, Tso A, Shankar V, Wendel BS, Bakerman I, Liang G, Koyano T, Fong R, Nau AN, Ahmad H, Gopakumar J, Wirka R, Lee AS, Boyd J, Woo YJ, Quertermous T, Gulati GS, Jaiswal S, Chien YH, Chan CKF, Davis MM, Nguyen PK. Human coronary plaque T cells are clonal and cross-react to virus and self. Circ Res 2022;130:1510–1530. 35430876 10.1161/CIRCRESAHA.121.320090 PMC9286288

[23] Alsaigh T , Evans D, Frankel D, Torkamani A. Decoding the transcriptome of atherosclerotic plaque at single-cell resolution. bioRxiv 2020:2020.2003.2003.968123. 10.1038/s42003-022-04056-7 PMC9556750 36224302

[24] Ma WF , Hodonsky CJ, Turner AW, Wong D, Song Y, Mosquera JV, Ligay AV, Slenders L, Gancayco C, Pan H, Barrientos NB, Mai D, Alencar GF, Owsiany K, Owens GK, Reilly MP, Li M, Pasterkamp G, Mokry M, van der Laan SW, Khomtchouk BB, Miller CL. Enhanced single-cell RNA-seq workflow reveals coronary artery disease cellular cross-talk and candidate drug targets. Atherosclerosis 2022;340:12–22. 34871816 10.1016/j.atherosclerosis.2021.11.025 PMC8919504

[25] Tannahill GM , Curtis AM, Adamik J, Palsson-McDermott EM, McGettrick AF, Goel G, Frezza C, Bernard NJ, Kelly B, Foley NH, Zheng L, Gardet A, Tong Z, Jany SS, Corr SC, Haneklaus M, Caffrey BE, Pierce K, Walmsley S, Beasley FC, Cummins E, Nizet V, Whyte M, Taylor CT, Lin H, Masters SL, Gottlieb E, Kelly VP, Clish C, Auron PE, Xavier RJ, O’Neill LA. Succinate is an inflammatory signal that induces IL-1beta through HIF-1alpha. Nature 2013;496:238–242. 23535595 10.1038/nature11986 PMC4031686

[26] Rubic T , Lametschwandtner G, Jost S, Hinteregger S, Kund J, Carballido-Perrig N, Schwarzler C, Junt T, Voshol H, Meingassner JG, Mao X, Werner G, Rot A, Carballido JM. Triggering the succinate receptor GPR91 on dendritic cells enhances immunity. Nat Immunol 2008;9:1261–1269. 18820681 10.1038/ni.1657

[27] Tan Z , Xie N, Cui H, Moellering DR, Abraham E, Thannickal VJ, Liu G. Pyruvate dehydrogenase kinase 1 participates in macrophage polarization via regulating glucose metabolism. J Immunol 2015;194:6082–6089. 25964487 10.4049/jimmunol.1402469 PMC4458459

[28] Gudi R , Bowker-Kinley MM, Kedishvili NY, Zhao Y, Popov KM. Diversity of the pyruvate dehydrogenase kinase gene family in humans. J Biol Chem 1995;270:28989–28994. 7499431 10.1074/jbc.270.48.28989

[29] Ostroukhova M , Goplen N, Karim MZ, Michalec L, Guo L, Liang Q, Alam R. The role of low-level lactate production in airway inflammation in asthma. Am J Physiol Lung Cell Mol Physiol 2012;302:L300–L307. 22080752 10.1152/ajplung.00221.2011 PMC3289274

[30] Min BK , Oh CJ, Park S, Lee JM, Go Y, Park BY, Kang HJ, Kim DW, Kim JE, Yoo EK, Kim HE, Kim MJ, Jeon YH, Kim YH, Lee CH, Jeon JH, Lee IK. Therapeutic effect of dichloroacetate against atherosclerosis via hepatic FGF21 induction mediated by acute AMPK activation. Exp Mol Med 2019;51:1–12. 10.1038/s12276-019-0315-2 PMC6802614 31570705

[31] Semba H , Takeda N, Isagawa T, Sugiura Y, Honda K, Wake M, Miyazawa H, Yamaguchi Y, Miura M, Jenkins DM, Choi H, Kim JW, Asagiri M, Cowburn AS, Abe H, Soma K, Koyama K, Katoh M, Sayama K, Goda N, Johnson RS, Manabe I, Nagai R, Komuro I. HIF-1alpha-PDK1 axis-induced active glycolysis plays an essential role in macrophage migratory capacity. Nat Commun 2016;7:11635. 27189088 10.1038/ncomms11635 PMC4873978

[32] Menk AV , Scharping NE, Moreci RS, Zeng X, Guy C, Salvatore S, Bae H, Xie J, Young HA, Wendell SG, Delgoffe GM. Early TCR signaling induces rapid aerobic glycolysis enabling distinct acute T cell effector functions. Cell Rep 2018;22:1509–1521. 29425506 10.1016/j.celrep.2018.01.040 PMC5973810

[33] Lee SJ , Jeong JY, Oh CJ, Park S, Kim JY, Kim HJ, Doo Kim N, Choi YK, Do JY, Go Y, Ha CM, Choi JY, Huh S, Ho Jeoung N, Lee KU, Choi HS, Wang Y, Park KG, Harris RA, Lee IK. Pyruvate dehydrogenase kinase 4 promotes vascular calcification via SMAD1/5/8 phosphorylation. Sci Rep 2015;5:16577. 26560812 10.1038/srep16577 PMC4642318

[34] Edsfeldt A , Duner P, Stahlman M, Mollet IG, Asciutto G, Grufman H, Nitulescu M, Persson AF, Fisher RM, Melander O, Orho-Melander M, Boren J, Nilsson J, Goncalves I. Sphingolipids contribute to human atherosclerotic plaque inflammation. Arterioscler Thromb Vasc Biol 2016;36:1132–1140. 27055903 10.1161/ATVBAHA.116.305675

[35] Deuse T , Hua X, Wang D, Maegdefessel L, Heeren J, Scheja L, Bolanos JP, Rakovic A, Spin JM, Stubbendorff M, Ikeno F, Langer F, Zeller T, Schulte-Uentrop L, Stoehr A, Itagaki R, Haddad F, Eschenhagen T, Blankenberg S, Kiefmann R, Reichenspurner H, Velden J, Klein C, Yeung A, Robbins RC, Tsao PS, Schrepfer S. Dichloroacetate prevents restenosis in preclinical animal models of vessel injury. Nature 2014;509:641–644. 24747400 10.1038/nature13232 PMC4323184

[36] Stacpoole PW . Therapeutic targeting of the pyruvate dehydrogenase complex/pyruvate dehydrogenase kinase (PDC/PDK) axis in cancer. J Natl Cancer Inst 2017;109. 10.1093/jnci/djx187 29059435

[37] Tiersma JF , Evers B, Bakker BM, Jalving M, de Jong S. Pyruvate dehydrogenase kinase inhibition by dichloroacetate in melanoma cells unveils metabolic vulnerabilities. Int J Mol Sci 2022;23:3745. 10.3390/ijms23073745 PMC8999016 35409102

[38] Michelakis ED , Gurtu V, Webster L, Barnes G, Watson G, Howard L, Cupitt J, Paterson I, Thompson RB, Chow K, O’Regan DP, Zhao L, Wharton J, Kiely DG, Kinnaird A, Boukouris AE, White C, Nagendran J, Freed DH, Wort SJ, Gibbs JSR, Wilkins MR. Inhibition of pyruvate dehydrogenase kinase improves pulmonary arterial hypertension in genetically susceptible patients. Sci Transl Med 2017;9:eaao4583. 10.1126/scitranslmed.aao4583 29070699

[39] Leow HW , Koscielniak M, Williams L, Saunders PTK, Daniels J, Doust AM, Jones MC, Ferguson GD, Bagger Y, Horne AW, Whitaker LHR. Dichloroacetate as a possible treatment for endometriosis-associated pain: a single-arm open-label exploratory clinical trial (EPiC). Pilot Feasibility Stud 2021;7:67. 10.1186/s40814-021-00797-0. 33712086 PMC7953373

[40] McCall CE , Zabalawi M, Liu T, Martin A, Long DL, Buechler NL, Arts RJW, Netea M, Yoza BK, Stacpoole PW, Vachharajani V. Pyruvate dehydrogenase complex stimulation promotes immunometabolic homeostasis and sepsis survival. JCI Insight 2018;3:e99292. 10.1172/jci.insight.99292 PMC6129136 30089711

[41] McCall CE , Zhu X, Zabalawi M, Long D, Quinn MA, Yoza BK, Stacpoole PW, Vachharajani V. Sepsis, pyruvate, and mitochondria energy supply chain shortage. J Leukoc Biol 2022;**112**: 1509–1514. 10.1002/JLB.3MR0322-692RR PMC9796618 35866365

[42] Ridker PM , Everett BM, Thuren T, MacFadyen JG, Chang WH, Ballantyne C, Fonseca F, Nicolau J, Koenig W, Anker SD, Kastelein JJP, Cornel JH, Pais P, Pella D, Genest J, Cifkova R, Lorenzatti A, Forster T, Kobalava Z, Vida-Simiti L, Flather M, Shimokawa H, Ogawa H, Dellborg M, Rossi PRF, Troquay RPT, Libby P, Glynn RJ, Group CT. Antiinflammatory therapy with canakinumab for atherosclerotic disease. N Engl J Med 2017;377:1119–1131. 28845751 10.1056/NEJMoa1707914

[43] Ridker PM , MacFadyen JG, Thuren T, Libby P. Residual inflammatory risk associated with interleukin-18 and interleukin-6 after successful interleukin-1beta inhibition with canakinumab: further rationale for the development of targeted anti-cytokine therapies for the treatment of atherothrombosis. Eur Heart J 2020;41:2153–2163. 31504417 10.1093/eurheartj/ehz542

[44] Everett BM , MacFadyen JG, Thuren T, Libby P, Glynn RJ, Ridker PM. Inhibition of interleukin-1beta and reduction in atherothrombotic cardiovascular events in the CANTOS trial. J Am Coll Cardiol 2020;76:1660–1670. 33004131 10.1016/j.jacc.2020.08.011

[45] Jha AK , Huang SC, Sergushichev A, Lampropoulou V, Ivanova Y, Loginicheva E, Chmielewski K, Stewart KM, Ashall J, Everts B, Pearce EJ, Driggers EM, Artyomov MN. Network integration of parallel metabolic and transcriptional data reveals metabolic modules that regulate macrophage polarization. Immunity 2015;42:419–430. 25786174 10.1016/j.immuni.2015.02.005

[46] Littlewood-Evans A , Sarret S, Apfel V, Loesle P, Dawson J, Zhang J, Muller A, Tigani B, Kneuer R, Patel S, Valeaux S, Gommermann N, Rubic-Schneider T, Junt T, Carballido JM. GPR91 Senses extracellular succinate released from inflammatory macrophages and exacerbates rheumatoid arthritis. J Exp Med 2016;213:1655–1662. 27481132 10.1084/jem.20160061 PMC4995082

[47] Hobert JA , Mester JL, Moline J, Eng C. Elevated plasma succinate in PTEN, SDHB, and SDHD mutation-positive individuals. Genet Med 2012;14:616–619. 22261759 10.1038/gim.2011.63 PMC4019996

[48] Cimen H , Han MJ, Yang Y, Tong Q, Koc H, Koc EC. Regulation of succinate dehydrogenase activity by SIRT3 in mammalian mitochondria. Biochemistry 2010;49:304–311. 20000467 10.1021/bi901627u PMC2826167

[49] Finley LW , Haas W, Desquiret-Dumas V, Wallace DC, Procaccio V, Gygi SP, Haigis MC. Succinate dehydrogenase is a direct target of sirtuin 3 deacetylase activity. PLoS One 2011;6:e23295. 10.1371/journal.pone.0023295 PMC3157345 21858060

[50] Li YH , Choi DH, Lee EH, Seo SR, Lee S, Cho EH. Sirtuin 3 (SIRT3) regulates alpha-smooth muscle actin (alpha-SMA) production through the succinate dehydrogenase-G protein-coupled receptor 91 (GPR91) pathway in hepatic stellate cells. J Biol Chem 2016;291:10277–10292. 26912655 10.1074/jbc.M115.692244 PMC4858976

[51] Stacpoole PW , Moore GW, Kornhauser DM. Metabolic effects of dichloroacetate in patients with diabetes mellitus and hyperlipoproteinemia. N Engl J Med 1978;298:526–530. 625308 10.1056/NEJM197803092981002

[52] Moore GW , Swift LL, Rabinowitz D, Crofford OB, Oates JA, Stacpoole PW. Reduction of serum cholesterol in two patients with homozygous familial hypercholesterolemia by dichloroacetate. Atherosclerosis 1979;33:285–293. 486225 10.1016/0021-9150(79)90180-1

[53] Stacpoole PW . The dichloroacetate dilemma: environmental hazard versus therapeutic goldmine–both or neither? Environ Health Perspect 2011;119:155–158. 20920954 10.1289/ehp.1002554 PMC3040600

[54] Khan AUH , Allende-Vega N, Gitenay D, Gerbal-Chaloin S, Gondeau C, Vo DN, Belkahla S, Orecchioni S, Talarico G, Bertolini F, Bozic M, Valdivielso JM, Bejjani F, Jariel I, Lopez-Mejia IC, Fajas L, Lecellier CH, Hernandez J, Daujat M, Villalba M. The PDK1 inhibitor dichloroacetate controls cholesterol homeostasis through the ERK5/MEF2 pathway. Sci Rep 2017;7:10654. 28878225 10.1038/s41598-017-10339-5 PMC5587676

