# Supplementary material for: Pyruvate dehydrogenase kinase regulates vascular inflammation in atherosclerosis and increases cardiovascular risk
Source: Cardiovasc Res. 2023 Mar 2;119(7):1524–36. doi: 10.1093/cvr/cvad038 (PMC10318388; doi:10.1093/cvr/cvad038)
Supplement: cvad038_Supplementary_Data [file cvad038_supplementary_data.docx]

**SUPPLEMENTARY MATERIAL**

**Pyruvate dehydrogenase kinase regulates vascular inflammation in atherosclerosis and increases cardiovascular risk**

***Short title: The role of PDK in atherosclerosis***

Maria J. Forteza^1^, Martin Berg^1^, Andreas Edsfeldt ^2,3,4^, Jangming Sun^2,3^, Roland Baumgartner^1^, Ilona Kareinen^1^, Felipe Beccaria Casagrande^1^, Ulf Hedin^5^, Song Zhang^6,7^, Ivan Vuckovic^6^, Petras P. Dzeja^7^, Konstantinos A. Polyzos^1^, Anton Gisterå^1^, Mette Trauelsen^8^, Thue W. Schwartz^8^, Lea Dib^9^, Joerg Herrmann^7^, Claudia Monaco9, Ljubica Matic^5^, Isabel Gonçalves^2,3^, and Daniel F.J. Ketelhuth^1,10^

**Correspondent author:**

Daniel FJ Ketelhuth, PhD

Department of Cardiovascular and Renal Research

University of Southern Denmark

J.B. Winsløws vej 21, 3

5000 Odense C

E-mail: ketelhuth@health.sdu.dk

**EXTENDED METHODS SECTION**

***Vulnerability index on CPIP carotid plaques***

A vulnerability index has been created to identify rupture-prone/high-risk plaque phenotypes ^1^. Previous work using the CPIP biobank showed that this index reflects well the delicate balance between stable and vulnerable plaques and could predict future cardiovascular events ^2^. Briefly, the vulnerability index is generated from eight-micrometre sections from the most stenotic part of the plaque, from 47 patients, which were used to stain for macrophages (CD68), smooth muscle cells (α-actin), intra-plaque haemorrhage (glycophorin A), neutral lipids (Oil Red O) and collagen (Russell-Movat Pentachrome), as previously described field^3, 4^. The stained plaque area of each component was analyzed using Biopix iQ 2.1.8 (Gothenburg, Sweden). The vulnerability index was calculated as a ratio between the sum of %ORO, %CD68, and %glycophorin A, and the sum of % α-actin and %collagen (all % of total plaque area).

***Gene set enrichment analysis (GSEA) – CPIP data***

CPIP mRNA-Seq data was used to perform a GSEA on the obtained Pearson correlation coefficients between PDK1, PDK2, PDK3, and PDK4 expression and collections of hallmark gene sets from the Molecular Signatures Database (MSigDB) ^5^. Normalized enrichment score (NES) and BH adjusted p-values were reported. GSEA was implemented using a R package “fgsea” (https://bioconductor.org/packages/release/bioc/html/fgsea.html).

***RNA sequencing of CPIP carotid plaques***

Human atherosclerotic plaque gene expression was collected from 78 plaques: 51 with symptoms <31 days and 27 without symptomatic carotid stenosis. RNA was extracted using Trizol and cleared of Ribosomal RNA using Ribo-ZeroTM Magnetic Kit from (Epicentre). RNA-Seq (strand-specific) libraries were prepared with ScriptSeqTM v2 RNA-Seq Library v2 Preparation Kit (Epicentre), as previously described ^6^. Samples were sequenced using the Illumina HiSeq2000 and the NextSeq platforms.

The spliced transcripts alignment to a reference (STAR)^7^ software was used to map short reads to human genome assembly GRCh38. Gene counts were then obtained and normalized by edgeR^8^. Batch effects of sequencing platforms were adjusted by an empirical Bayes method ^9^ resulting in gene expressions as log2- transformed count per million (CPM).

***Analyses of public datasets of scRNAseq using PlaqView***

PlaqView ([www.plaqview.com](http://www.plaqview.com)) is a standalone, interactive, and reproducible web-based tool to explore single-cell RNA-sequencing data of cardiovascular tissue material. We used the PlaqView gene expression query to evaluate the expression of PDK isoforms in major cells population from human atherosclerotic plaques. PlaqView is built on R and Shiny, and its source code is publicly available at <https://github.com/MillerLab-CPHG/PlaqView>.

***Human atherosclerotic plaque proteomics data - BiKE***

Human carotid endarterectomy samples from eighteen patients, matched for the male sex, age and statin medication, were used for proteomics analysis as described^10^. Briefly, freshly collected samples were dissected in two parts, namely, a central portion corresponding to the maximum stenosis and the respective adjacent tissue, apparently with no disease, were snap frozen. Samples were then crushed while frozen using a tissue pulverizer (Cellcrusher, Cork, Ireland) and lysed and sonicated in a buffer containing 4% sodium dodecyl sulfate, 25 mmol/l N-2-hydroxyethylpiperazine-N0-2-ethane sulfonic acid, pH 7.6. Lysates were centrifuged and supernatants were collected for digestion. Digestion was performed, first with Lys-C (1:50 ratio) overnight and then with another round of trypsin digestion (1:50 ratio) again overnight. The resulting peptide mixtures were labelled with isobaric Tandem mass tags (TMT) (TMT10, Thermo Fisher Scientific, Waltham, Massachusetts). Samples were divided into 4 TMT10 sets, 2 containing the central samples and 2 containing the adjacent samples. Each set contained 9 samples, and the last channel contained an internal standard composed of peptides from both adjacent and central samples. After sample clean-up by solid-phase extraction (SPE strata-X-C, Phenomenex, Torrance, California), the sample pools were pre-fractionated by high-resolution isoelectric focusing and the resulting fractions were analyzed by LC-MS/MS ^11^. The fragment spectra from the mass spectrometer were matched to a database consisting of theoretical fragment spectra from all human proteins and filtered at a 1% false discovery rate on the peptide level to obtain protein identities (UniProt). Quantitative information was acquired by using the TMT reporter ion intensities.

***Immunofluorescence staining of human plaques - BiKE***

Carotid plaques from the BiKE biobank were fixed in a 4% buffered formaldehyde solution, dehydrated in a series of graded alcohol solutions, and embedded in paraffin wax. Sections of 5 µm were deparaffinized in Tissue Clear and rehydrated in graded ethanol solutions. All immunofluorescence reagents were from Biocare Medical, CA, USA unless otherwise specified. Antigen retrieval was performed with DIVA buffer. The tissue sections were stained with primary antibodies against PDK1 (Genetex, CA, US), PDK2 and PDK3 (Novus Biologicals, UK), PDK4 (Thermofisher, Sweden), CD68 and alpha-smooth muscle-actin (αSMA; Dako, Denmark) and CD3 (Biocare Medical, CA, USA). Detection was carried out with fluorescent secondary antibodies against rabbit or mouse (Vector Laboratories, CA, USA) and nuclei were counterstained with DAPI (Thermofisher, CA, USA).

***Western blot analyses of PDH phosphorylation in BiKE carotid plaque protein extracts***

Human carotid endarterectomy samples from eighteen patients from BiKE, matched for the male sex, age and statin medication, as previously described ^10^, were used for the quantification of PDH pSer233, pSer293, (rabbit anti-mouse/human; Merk-Millipore, Darmstadt, Germany) and unphosphorylated PDH by Western Blot (rabbit anti-mouse/human Abcam, Cambridge, UK). Briefly, freshly collected samples were dissected and snap-frozen. Samples were then crushed while frozen using a tissue pulverizer (Cellcrusher, Cork, Ireland) and lysed and sonicated in a buffer containing 4% sodium dodecyl sulfate, 25 mmol/L N-2-hydroxyethylpiperazine-N0-2-ethane sulfonic acid, pH 7.6. Lysates were centrifuged and supernatants were collected for Western blot analysis.

***Ex vivo culture of cells isolated from human carotid atherosclerotic plaques***

Fresh diseased intimal arterial segments, taken from patients undergoing revascularization procedures for symptomatic carotid disease, were dissected from carotid endarterectomy specimens under a dissecting microscope. Single-cell suspensions were obtained by enzymatic digestion, as previously published ^12^. In brief, freshly isolated atheroma cells were cultured at 1×10^6^ cells/ml in RPMI containing 10% fetal bovine serum in at least three replicate wells (Biosera, Ringmer, UK). Viability was determined via Trypan Blue exclusion and propidium iodide (PI) after cell isolation and was > 95% in all preparations. Viability in culture was monitored with 3-(4,5-dimethyl-2-yl)-2,5-diphenyltetrazolium (Sigma, Dorset, UK). The isolated cells comprise a mixed population representing the major cell types

resident in human atherosclerotic plaques (mostly macrophages, SMCs and T lymphocytes),

which spontaneously produce a wide range of pro-inflammatory mediators without extrinsic

stimulation. The cells are not passaged nor stimulated, but placed in culture for 24-28 hours

during which maintain a composition of CD3+lymphocytes (6–15% in different

preparations), CD68+ macrophages (30–44%), and smooth muscle cells (10–20%), and have

a viability of 95-98% upon isolation. This model offers the ability to dissect the role of

endogenous individual molecules from the human atheroma micro-environment^13^. Supernatants were removed after 24 h and stored at –80 °C for single-batch analysis. ELISA was used to quantify levels of cytokines in the supernatant (Millipore Corporation, MA, USA). Each replicate culture was analyzed in duplicate.

***Mouse tissue processing, immunohistochemistry, and lesion analysis***

After sacrifice, vascular perfusion was performed with sterile ribonuclease-free PBS. The aortic arch was fixed in a PBS-buffered 4% formaldehyde solution for subsequent pinning and staining with Sudan IV (Sigma-Aldrich, St. Louis, USA). The plaque area was calculated as the percentage of the total surface area of the thoracic aorta. The rest of the aorta was dissected and snap-frozen for subsequent RNA or protein isolation. The heart was dissected and preserved in OCT compound for immunohistochemistry. Lesion analysis was performed as previously described ^14^. In brief, the hearts were serially sectioned on a cryostat, starting from the proximal part of the aortic root, and stained with hematoxylin and Oil Red O. Lesion size was determined on eight sections collected every 100 μm along the aortic root. For each section, images were captured in a Leica photomicroscope, and the surface areas of the lesions and the entire vessel were measured using ImageJ software (NIH). Inflammatory cell markers in aortic root sections were evaluated using primary antibodies against vascular cell adhesion molecule 1 (VCAM-1) (Abcam, Cambridge, UK); CD68 (AbD Serotec, Oxford, UK); αSMA (Abcam, Cambridge, UK); FOXP3 (Abcam, Cambridge, UK); and CD4 (BD Biosciences, New Jersey, USA) that were applied to acetone-fixed cryosections. Detection was performed using an ABC horseradish peroxidase kit (Vector Laboratories, Burlingame, USA) as previously described ^14^. Collagen content was assessed by analyzing formaldehyde-fixed sections stained for 1 h in saturated picric acid containing 0.1% picrosirius red (Fluka, Buchs, Switzerland) and visualized under linearly polarized light as previously described^15^. Necrotic cores were detected by routine H&E staining of aortic root frozen sections [Iron hematoxylin (Weigert's) kit for histology (Sigma-Aldrich, St. Louis, USA); Eosin 0.2% (Histolab, Gothenburg, Sweden)].

Samples that were compromised during processing or analysis were excluded from the study. Assessment of samples was coded, and the evaluation performed by trained persons, which were blinded to the treatment groups.

***Mouse blood analyses***

Blood was collected by cardiac puncture in EDTA-coated tubes at the endpoint. Whole blood was analyzed on a Vet ABC hemocounter (Scil Animal Care, Viernheim, Germany). Plasma levels of cholesterol, triglycerides and lactate were analyzed using enzymatic colorimetry (Randox, Crumlin, UK).

***Hepatic cholesterol levels***

Liver lipids were extracted from liver samples using the Folch method ^16^. Briefly, lysates were homogenized in methanol, and lipids were extracted by chloroform separation (methanol: chloroform (1:2)). After drying, the extracts were redissolved in 1% Triton-100, and cholesterol content was measured using enzymatic colorimetric kits (Randox Lab. Ltd. Crumlin, UK) according to the manufacturer’s instructions.

***Quantitative PCR***

RNA was isolated using a RNeasy kit (Qiagen, Hilden, Germany), reverse-transcribed, and amplified by real-time PCR using Assay-On-Demand primers and probes (Applied Biosystems, MA, USA; listed in Supplementary table 3). Hypoxanthine-guanine phosphoribosyltransferase was used as a housekeeping gene. The relative expression was calculated using the formula 2^−ΔΔCt^, where ΔCt is the Ct of the housekeeping gene is subtracted from the target gene Ct, and ΔΔCt = ΔCt (sample) − ΔCt (calibrator = average ΔCt of the control group).

***Western blot analyses of the inflammasome activation***

Total protein was extracted from frozen mouse aorta samples. Protein extracts (50-70 µg) were separated by SDS–PAGE (4–15%, Bio-Rad Laboratories, CA, USA) and transferred to PVDF membranes (GE Healthcare, Uppsala, Sweden). The membranes were probed for murine Caspase 1, IL-1β, and Vinculin (all from Abcam, Cambridge, UK) that was used as a loading control.

***In vitro proliferation of splenocytes and cytokine secretion***

Splenocytes from control and DCA-treated mice were incubated in duplicate in anti-CD3- and anti-CD28-coated 96-well plates. Briefly, 1 μCi H3-thymidine (Sigma-Aldrich, St. Louis, USA) was added after 60 h, and DNA replication was measured with a scintillation counter. The results are expressed as counts per minute (CPM). The secretion of cytokines, including interleukin (IL)-10, IFN-γ, and IL-17, by proliferating splenocytes were analyzed in the supernatants of cultures by ELISA (R&D Systems, Minneapolis, USA) according to the manufacturer’s instructions.

***Flow cytometry analysis***

Flow cytometry was performed on leukocytes isolated as single-cell suspensions from the spleen. Fixable Aqua Live/Dead staining was used according to the manufacturer’s protocol (Invitrogen). After Fc-block incubation (anti-CD16/32, BD Biosciences, New Jersey, USA), the following fluorophore-labelled primary IgG antibodies were used for extracellular staining: F4/80, CD11b, CD11c, CD206, CD4, and CD25 (all from BD Biosciences, New Jersey, USA). Intracellular staining was performed using a Foxp3 staining set (eBioscience, San Diego, USA) and mouse RORγ (BD Biosciences, New Jersey, USA). Samples were acquired on a CyAn ADP flow cytometer (Dako, Næstved, Denmark), and the data were analyzed using FlowJo software (TreeStar software, Ashland, USA). The gating strategy for spleen cells is shown in Supplementary Fig 8.

***Metabolomic analysis of mouse aortas***

Metabolite levels in the aorta samples were analyzed by ^1^H-NMR spectroscopy.

Tissues were ground under liquid nitrogen using a mortar and pestle. Water-soluble metabolites were extracted in ice-cold 6% perchloric acid (PCA). Deproteinized extracts were transferred to ice-cold centrifuge tubes and centrifuged at 10,000 g for 10 minutes at 4 °C. The supernatant was transferred to fresh cold centrifuge tubes and neutralized to pH 7 with 2M KHCO_3_. After neutralization, the tissue extracts were centrifuged; the supernatant was collected and 250 μL was reconstructed by adding 150 μL of 0.1 M phosphate buffer and 50 μL of 1 mM TSP-*d*4 solution in D_2_O. Samples were vortexed for 20 seconds and transferred to 5 mm NMR tubes. NMR spectra were acquired on a Bruker 500 MHz Avance III HD spectrometer equipped with a BBO cryoprobe and SampleCase autosampler (Bruker Biospin, Rheinstetten, Germany). ^1^H-NMR spectra were recorded using 1D noesy pulse sequence with presaturation (noesygppr1d), with a 90-degree pulse (~13 µs), 4.68 seconds acquisition time, and 4 seconds relaxation delay. Spectra were phase and baseline corrected using the Topspin 3.5 software. Metabolites were identified and quantified using the software program Chenomx NMR Suite 8.2.

***Murine bone marrow-derived macrophage (BMDM) generation***

Bone marrow cells from 8-week-old male C57BL/6J and GPR91KO mice were isolated from the hind leg bones (femur and tibia) and differentiated into bone marrow-derived macrophage (BMDM) in DMEM (containing 10% fetal calf serum, 1% penicillin/streptomycin and 20% L929 supernatant) for 6 days, at which time they were counted and replated for experiments. BMDMs (0.5x10^6^ BMDMs/mL) were used for *in vitro* experiments. All animal experiments were conducted according to the guidelines of Directive 2010/63/EU of the European Parliament on the protection of animals used for scientific purposes and approved by the Ethical Committee of the Northern Stockholm region board (N28-15). GPR91KO mice were generously shared by Dr. José M. Carballido, previously described^17^.

***Evaluation of the inflammasome activation in macrophages in vitro***

To prime the NLRP3 inflammasome, 5x10^5^ BMDMs were pretreated with 10 ng/mL LPS (Enzo Biochem, Farmingdale, NY, USA) for 4 h, followed by the addition of 1 mmol/L ATP (Sigma-Aldrich, St. Louis, IL, USA) for 1 h. Additionally, some cells were pretreated with DCA to inactivate inflammasome activity for 30 min and then treated with LPS and/or ATP. Similarly, some cells were activated with 10 ng/mL LPS and then treated with DCA for 30 min before adding 1 mmol/L ATP for 1 h. In addition, some cells were treated with different concentrations (0, 5, or 10 mmol/L) of sodium succinate or with dimethyl succinate (Sigma-Aldrich, St. Louis, USA) with or without pertussis toxin (200 ng/mL, Sigma-Aldrich, St. Louis, USA), and with or without GPR91 antagonist validated at the CBMR Metabolic Receptology laboratory [cpmd0441 (IC_50_=2.63E-06 M); synthesized by SIA Enamine, Latvia] for 2 h prior to experiments. IL-1β production in the supernatants of cultures was analyzed by ELISA (R&D Systems, MN, USA) according to the manufacturer’s instructions.

***Mitochondrial ROS (mtROS) production***

THP-1 cells were differentiated into macrophages in a 96-well culture plate, with PMA (100 nM) overnight, and then the medium (RPMI containing 10% fetal calf serum, 1% penicillin/streptomycin) was changed. After 48h washout, THP-1 macrophages were pretreated with 10 ng/mL LPS (Enzo Biochem, Farmingdale, NY, USA) for 4 h, followed by the addition of 1 mmol/L ATP (Sigma-Aldrich, St. Louis, IL, USA) for 1 h, concomitantly or not with 10 mM DCA or 500 μM sodium succinate (both from Sigma-Aldrich, IL, USA). Mitochondria-associated ROS levels were measured by staining cells with MitoSOX (Fisher Scientific, CA, USA) at 2.5mM for 15 min at 37 ºC, and fluorescence detection using FlexStation reader (Molecular Devices, CA, USA) according to the manufacturer’s instructions (excitation = 510 nm, emission = 580 nm).

***siRNA transfection***

PMA-differentiated THP-1 macrophages were transfected with 500 nM of control, PDK1, PDK2, PDK3, and PDK4 siRNA (Dharmacon, Lafayette, CO) with DharmaFECT 1 transfection reagent (Dharmacon, Lafayette, CO) according to the manufacturer’s protocol. The siRNA inhibition efficiency was quantified by qPCR using TaqMan assay-on-demand primers and probes (Thermofisher, CA, USA).

**SUPPLEMENTARY FIGURES**


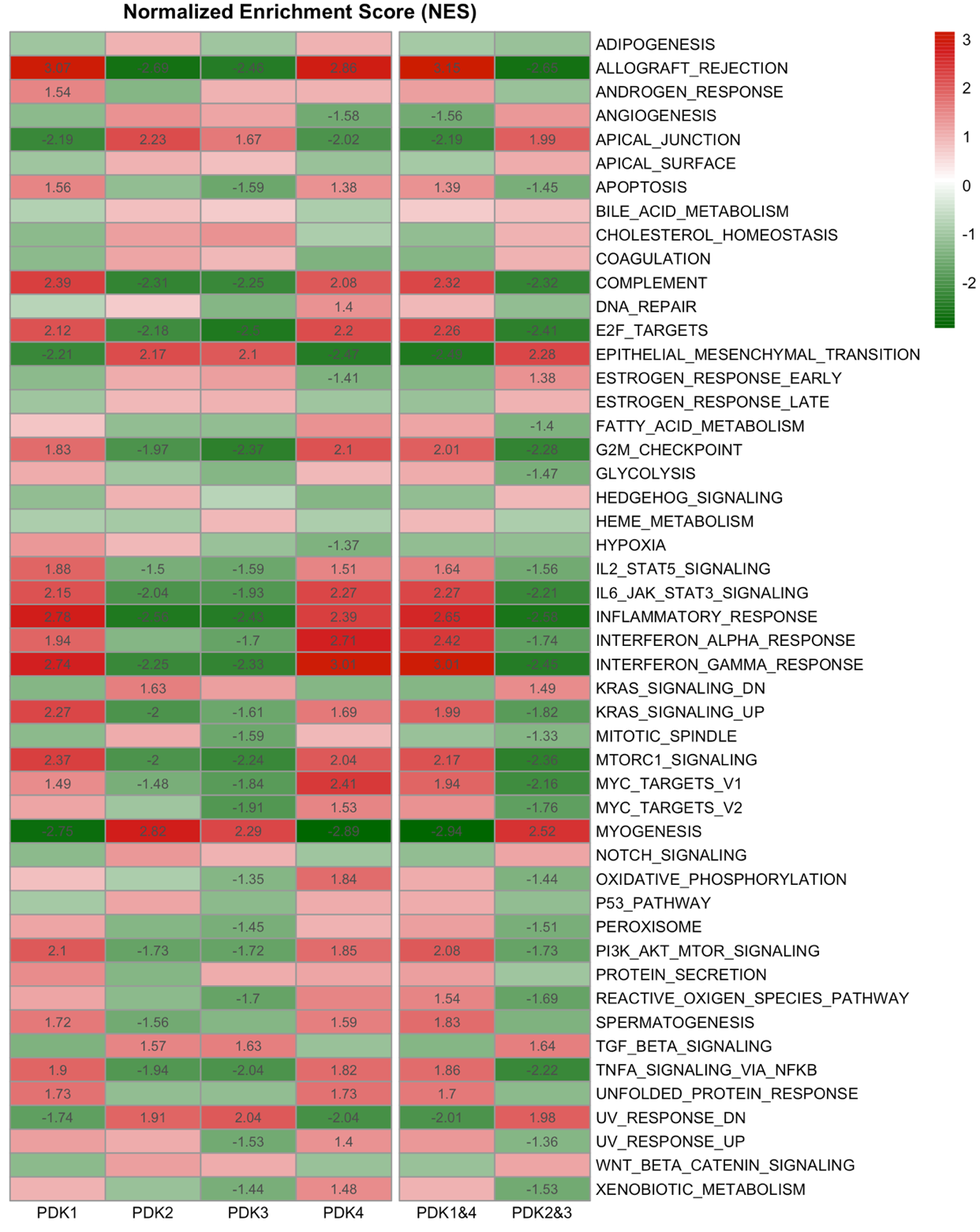

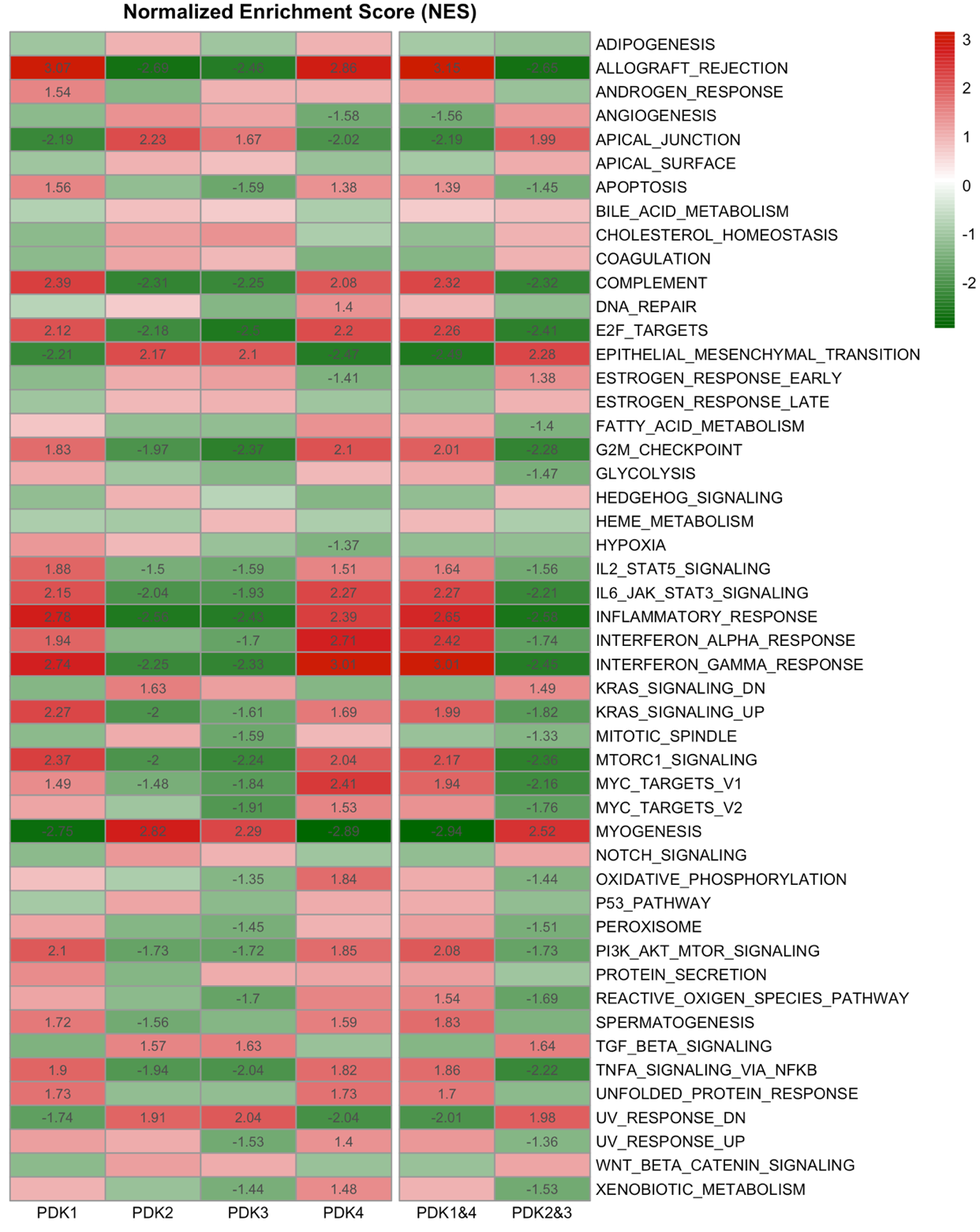


**Supplementary Fig.1. Gene set enrichment analysis (GSEA) on respective PDK1-4 correlated pathways.**

Restricted to hallmark gene sets, the number of the normalized enrichment score (NES) is displayed if adjusted p-values < 0.05. A positive/negative NES represents that most genes in this pathway are positive/negatively correlated with PDK1-4. Hallmark gene sets were defined by the Molecular Signatures Database (MSigDB) ^5^.

**Supplementary Fig.2. Expression of PDK isoforms in human atherosclerotic plaques of asymptomatic and symptomatic patients.**

Gene expression analysis of PDKs isoforms in the corresponding carotid plaques of symptomatic (S) and asymptomatic (AS) patients; n = 78. (B) Gene expression analysis of PDKs isoforms 2&3 and isoforms 1&4 corresponding to carotid plaques of symptomatic (S) and asymptomatic (AS) patients; n = 78.

**Supplementary Fig.3. Evaluation of lipid- and inflammatory-related readouts in the liver of DCA-treated and control mice.**

(A) Hepatic cholesterol levels (n = 7 and 8 for controls and DCA-treated groups, respectively). (B) mRNA quantification of lipid metabolism-related genes in the liver (n = 10 and 12 for control and DCA treated groups, respectively); Mann-Whitney U-test analysis. (C) Correlation between hepatic *Ldlr* mRNA levels and plasma cholesterol at the endpoint; linear regression analysis (n = 10 and 12 for controls and DCA treated groups, respectively). (C) Correlation between hepatic *Ldlr* mRNA levels and plasma triglycerides at the endpoint; linear regression analysis (n = 10 and 12 for controls and DCA treated groups, respectively). (E-F) mRNA quantification of innate- (left graph) and adaptive immune (right graph)-related genes in the liver (n = 10 and 12 for control and DCA treated groups, respectively); Mann-Whitney U-test analysis. (A-F) 1 mg/ml (~170 mg/Kg/day) DCA. The results show the mean ± SEM and are polled data from 2 independent experiments. *Srebf 1-2*, Sterol Regulatory Element Binding Transcription Factor 1-2; *Fas*, fatty acid synthase; *Hmgcoas*, HMG-CoA synthase; *Hmgcoar*, HMG-CoA reductase; *Ldlr*, LDL receptor; *Abca1*, ATP Binding Cassette Subfamily A Member 1; Lpl, lipoprotein lipase; Cyp7a1, cholesterol 7-alpha-monooxygenase-1; *Arg1*, arginase-1; *Chil3*, chitinase-like 3; *Tbet*, T-box expressed in T cells; *Gata3*, GATA Binding Protein 3; *Rorgt*, RAR-related orphan receptor gamma; *Foxp3*, forkhead box P3; *Ifng*, interferon-gamma. ^#^P= 0.056, *P < 0.05.

**Supplementary Fig.4. Blood cell counts in DCA-treated and control mice.**

Percentage of lymphocytes (Lym), monocytes (Mono), and granulocytes (Gran) in the blood of DCA-treated (1 mg/ml; ~170 mg/Kg/day) and control mice (n = 12 and 8, respectively); no significant differences were observed, Mann-Whitney U-test analysis. The results show the mean ± SEM and are pooled data from 2 independent experiments.

**Supplementary figure 5. PDK isoform correlations with CD68 content in plaques of DCA-treated mice and controls.**

Correlation between mRNA levels of PDK1 (top left), PDK2 (bottom left), PDK3 (top right), and PDK4 (bottom right) and % CD68 staining in plaques from of DCA-treated (1 mg/ml; ~170 mg/Kg/day) and control mice (n = 13); ‘r’ coefficient and P value defined by Linear regression analysis are polled data from 2 independent experiments.


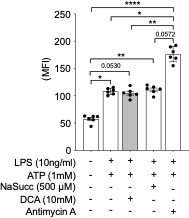


A

B

**Supplementary figure 6. Quantification of ROS production in THP-1 differentiated macrophages and of cell viability evaluation of BMDMs.**

(A) Mitochondria-associated ROS levels (MitoSOX) in THP-1 differentiated macrophages pretreated with 10 ng/mL LPS for 4 h, followed by the addition of 1 mmol/L ATP for 1 h, concomitantly or not with 10 mM DCA or 500 μM sodium succinate, or the positive control Antimyxin A; n = 6, pooled data from 2 experiments, in triplicate wells. (B) Lactate dehydrogenase (LDH) release by BMDMs unstimulated and stimulated with LPS (10 ng/mL), in the presence and absence of DCA (30 mM). (A-B) Kruskal-Wallis ANOVA with Dunn’s post-test analysis. *P < 0.05, **P < 0.01, ***P < 0.001, ****P<0,00001.

**Supplementary figure 7: IL1-b release by BMDM.**

(A) Quantification of IL-1β secretion by BMDM pre-treated 1h with Diethyl Succinate before NLRP3 inflammasome priming and activation (n = differentiated cells from 6 individual mice, treated in duplicates); Kruskal-Wallis ANOVA with Dunn’s post-test analysis. (B) Quantification of IL-1β secretion after NLRP3 inflammasome activation on BMDM from WT and SUCNR1 KO (GPR91KO) mice (n = differentiated cells from 4 WT and 6 HPR91KO mice, treated in duplicates); Mann-Whitney U-test analysis.

**
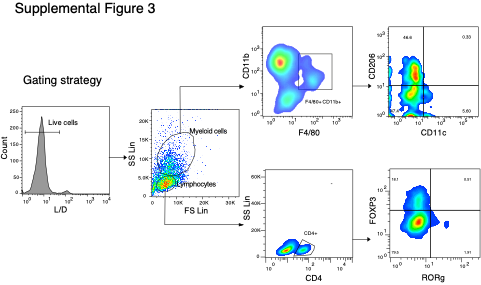
**

**Supplemental figure 8: Gating strategy for analysing macrophages (CD11b+ F4/80+) and CD4 T regulatory (CD4+ FOXP3+) or Th17 (CD4+ RORγ+) cells from spleen cells.**

**SUPPLEMENTARY TABLES**

**Supplementary table 1: Kinetics of body weight gain and plasma lipid levels.**

|  | Time of treatment | Control | 0.1 mg/ml | 1 mg/ml |
| --- | --- | --- | --- | --- |
| Body weight (mg) | 0 | 25.6±1.5 | 25.8±2.0 | 25.8±2.0 |
|  | 2 weeks | 27.1±1.0 | 28.8±2.9 | 28.8±2.9 |
|  | 4 weeks | 29.5±1.9 | 29.3±1.5 | 29.3±1.5 |
|  | 6 weeks | 31.2±2.3 | 30.2±1.6 | 30.2±1.6 |
|  | 8 weeks | 31.8±2.9 | 34.5±3.9 | 34.5±3.9 |
| Cholesterol (mg/ml) | 0 | 309.6±41.7 | 295.0±66.7 | 310.3±80.6 |
|  | 2 weeks | 415.6±99.2 | 393.9±74.2 | 406.8±91.5 |
|  | 4 weeks | 497.7±123.4 | 425.4±88.1 | 461.4±112.6 |
|  | 6 weeks | 625.2±199.7 | 598.8±114.5 | 573.5±182.6 |
|  | 8 weeks | 861.4±179.9 | 761.48±138±5 | 676.1±136.9** |
| Triglycerides (mg/ml) | 0 | 182.1±41.5 | 241.4±70.8 | 238.4±49.4 |
|  | 2 weeks | 173.5±38.2 | 220.5±113.6 | 244.8±24.2 |
|  | 4 weeks | 324.2±56.9 | 280.1±91.0 | 319.5±46.5 |
|  | 6 weeks | 416.5±127.3 | 323.4±149.8 | 386.2±117.2 |
|  | 8 weeks | 451.5±137.4 | 407.7±124.0 | 516.0±159.5 |

Data are mean ± SEM. 2-tailed 1-way ANOVA with Dunn’s post-test was performed for statistical analysis; **P < 0.01, control vs DCA (1mg/ml; ~170 mg/Kg/day).

**Supplementary table 2: Concentrations of metabolites in murine aortas**

| **Metabolite (µM)** | **Control** | **DCA 1mg/ml** | **P value** |
| --- | --- | --- | --- |
| ADP | 1.9±0.3 | 1,2±0,5 | 0.06 |
| AMP | 5.4±2 | 3,6±1,9 | 0.22 |
| Acetate | 8.7±0.8 | 7,2±0,9 | 0.03* |
| Acetone | 6.5±3.5 | 4±3,7 | 0.34 |
| Alanine | 11±2.5 | 9,5±1,7 | 0.31 |
| Aspartate | 4±1.1 | 3,4±0,9 | 0.38 |
| Betaine | 0.7±0.1 | 0,6±0,3 | 0.66 |
| Carnitine | 0.8±0.4 | 0,9±0,5 | 0.66 |
| Choline | 2.9±2.6 | 5,3±4,3 | 0.36 |
| Citrate | 3.9±1.3 | 3,5±0,5 | 0.63 |
| Creatine | 22.9±5.5 | 22,7±8,7 | 0.97 |
| Creatine phosphate | 2.1±0.6 | 1,8±0,2 | 0.53 |
| Formate | 7.7±1.6 | 5,7±1,1 | 0.06 |
| Fumarate | 0.6±0.1 | 0,5±0,2 | 0.58 |
| Glucose | 34.9±20.7 | 22,8±7,2 | 0.26 |
| Glutamate | 8.6±1.7 | 8,4±1,2 | 0.85 |
| Glutamine | 8.6±0.9 | 7,4±1,6 | 0.21 |
| Glutathione | 1.9±0.3 | n.d. | - |
| Glycerol | 9.5±5.8 | 12±9,2 | 0.66 |
| Glycine | 11.9±3.1 | 11,2±1,2 | 0.65 |
| Hypoxanthine | 1.7±0.8 | 1,7±1,1 | 0.95 |
| IMP | 1.5±0.8 | 1,7±0,7 | 0.70 |
| Inosine | 2.4±0.6 | 3,6±1,4 | 0.16 |
| Isoleucine | 1±0.1 | 0,7±0,1 | 0.004* |
| Lactate | 104.3±19.4 | 74,8±5,6 | 0.03* |
| Leucine | 1.4±0.2 | 1±0,1 | 0.004* |
| Lysine | 4.9±1.5 | 4±1,2 | 0.35 |
| Malate | 4.7±1.3 | 4±1,3 | 0.45 |
| Methanol | 143.3±8.7 | 146,3±4,4 | 0.51 |
| NAD+ | n.d. | 1,4±0,6 | - |
| Niacinamide | n.d. | 1,9±0,6 | - |
| O-Phosphocholine | 1.1±0.3 | 1±0,2 | 0.55 |
| Pyruvate | 1.1±0.5 | 1±1 | 0.92 |
| Succinate | 3.6±0.4 | 2,6±0,3 | 0.002* |
| Taurine | 73±11.2 | 68,5±14,6 | 0.62 |
| Threonine | 3.5±1.1 | 2,8±0,6 | 0.28 |
| Trimethylamine N-oxide | 1.3±0.7 | 0,9±0,2 | 0.34 |
| Tyrosine | 1.4±0.2 | 1,1±0,5 | 0.38 |
| Valine | 1.9±0.3 | 1,5±0,2 | 0.03* |
| myo-Inositol | 8±0.3 | 8,7±1,3 | 0.33 |
| sn-Glycero-3-phosphocholine | 2±0.5 | 1,4±1 | 0.38 |

(*) P< 0.05

**Supplementary table 3. List of Assay-on-demand primers/probes used in the study**

| **Gene target** | **Catalog number** |
| --- | --- |
| *Cd80* | Mm00711660_m1 |
| *Chil3* | Mm00657889_m1 |
| *Cxcl10* | Mm99999072_m1 |
| *Fizz1(RETNLA)* | Mm00445109_m1 |
| *Foxp3* | Mm00475162_m1 |
| *Gata3* | Mm00484683_m1 |
| *Hmox1* | Mm00516005_m1 |
| *Ifng* | Mm01168134_m1 |
| *IL1b* | Mm00434228_m1 |
| *Il6* | Mm00446190_m1 |
| *Il10* | Mm01288386_m1 |
| *Il12* | Mm00434169_m1 |
| *Il18* | Mm00434226_m1 |
| *Pdk1* | Mm00554300_m1 |
| *Pdk2* | Mm00446681_m1 |
| *Pdk3* | Mm00455220_m1 |
| *Pdk4* | Mm01166879_m1 |
| *Rorg* | Mm01261022_m1 |
| *Socs3* | Mm00545913_s1 |
| *Tbx21* | Mm00450960_m1 |
| *Tgfb* | Mm00441726_m1 |
| *Tnfa* | Mm00443258_m1 |

Assay-On-Demand primers and probes (Applied Biosystems, MA, USA)

**SUPPLEMENTARY** References

1. Erlov T, Cinthio M, Edsfeldt A, Segstedt S, Dias N, Nilsson J, Goncalves I. Determining carotid plaque vulnerability using ultrasound center frequency shifts. *Atherosclerosis* 2016;**246**:293-300.

2. Goncalves I, Sun J, Tengryd C, Nitulescu M, Persson AF, Nilsson J, Edsfeldt A. Plaque Vulnerability Index Predicts Cardiovascular Events: A Histological Study of an Endarterectomy Cohort. *J Am Heart Assoc* 2021;**10**:e021038.

3. Goncalves I, Moses J, Dias N, Pedro LM, Fernandes e Fernandes J, Nilsson J, Ares MP. Changes related to age and cerebrovascular symptoms in the extracellular matrix of human carotid plaques. *Stroke* 2003;**34**:616-622.

4. Tomas L, Edsfeldt A, Mollet IG, Perisic Matic L, Prehn C, Adamski J, Paulsson-Berne G, Hedin U, Nilsson J, Bengtsson E, Goncalves I, Bjorkbacka H. Altered metabolism distinguishes high-risk from stable carotid atherosclerotic plaques. *Eur Heart J* 2018;**39**:2301-2310.

5. Liberzon A, Birger C, Thorvaldsdottir H, Ghandi M, Mesirov JP, Tamayo P. The Molecular Signatures Database (MSigDB) hallmark gene set collection. *Cell Syst* 2015;**1**:417-425.

6. Edsfeldt A, Goncalves I, Grufman H, Nitulescu M, Duner P, Bengtsson E, Mollet IG, Persson A, Nilsson M, Orho-Melander M, Melander O, Bjorkbacka H, Nilsson J. Impaired fibrous repair: a possible contributor to atherosclerotic plaque vulnerability in patients with type II diabetes. *Arterioscler Thromb Vasc Biol* 2014;**34**:2143-2150.

7. Dobin A, Davis CA, Schlesinger F, Drenkow J, Zaleski C, Jha S, Batut P, Chaisson M, Gingeras TR. STAR: ultrafast universal RNA-seq aligner. *Bioinformatics* 2013;**29**:15-21.

8. Robinson MD, McCarthy DJ, Smyth GK. edgeR: a Bioconductor package for differential expression analysis of digital gene expression data. *Bioinformatics* 2010;**26**:139-140.

9. Johnson WE, Li C, Rabinovic A. Adjusting batch effects in microarray expression data using empirical Bayes methods. *Biostatistics* 2007;**8**:118-127.

10. Perisic L, Aldi S, Sun Y, Folkersen L, Razuvaev A, Roy J, Lengquist M, Akesson S, Wheelock CE, Maegdefessel L, Gabrielsen A, Odeberg J, Hansson GK, Paulsson-Berne G, Hedin U. Gene expression signatures, pathways and networks in carotid atherosclerosis. *J Intern Med* 2016;**279**:293-308.

11. Branca RM, Orre LM, Johansson HJ, Granholm V, Huss M, Perez-Bercoff A, Forshed J, Kall L, Lehtio J. HiRIEF LC-MS enables deep proteome coverage and unbiased proteogenomics. *Nat Methods* 2014;**11**:59-62.

12. Shalhoub J, Viiri LE, Cross AJ, Gregan SM, Allin DM, Astola N, Franklin IJ, Davies AH, Monaco C. Multi-analyte profiling in human carotid atherosclerosis uncovers pro-inflammatory macrophage programming in plaques. *Thromb Haemost* 2016;**115**:1064-1072.

13. Monaco C, Andreakos E, Kiriakidis S, Mauri C, Bicknell C, Foxwell B, Cheshire N, Paleolog E, Feldmann M. Canonical pathway of nuclear factor kappa B activation selectively regulates proinflammatory and prothrombotic responses in human atherosclerosis. *Proc Natl Acad Sci U S A* 2004;**101**:5634-5639.

14. Gistera A, Ketelhuth DF. Immunostaining of Lymphocytes in Mouse Atherosclerotic Plaque. *Methods Mol Biol* 2015;**1339**:149-159.

15. Ovchinnikova O, Robertson AK, Wagsater D, Folco EJ, Hyry M, Myllyharju J, Eriksson P, Libby P, Hansson GK. T-cell activation leads to reduced collagen maturation in atherosclerotic plaques of Apoe(-/-) mice. *Am J Pathol* 2009;**174**:693-700.

16. Folch J, Lees M, Sloane Stanley GH. A simple method for the isolation and purification of total lipides from animal tissues. *J Biol Chem* 1957;**226**:497-509.

17. Rubic T, Lametschwandtner G, Jost S, Hinteregger S, Kund J, Carballido-Perrig N, Schwarzler C, Junt T, Voshol H, Meingassner JG, Mao X, Werner G, Rot A, Carballido JM. Triggering the succinate receptor GPR91 on dendritic cells enhances immunity. *Nat Immunol* 2008;**9**:1261-1269.
